# Bioluminescent Intercalating Dyes for Ratiometric Nucleic Acid Detection

**DOI:** 10.1021/acschembio.3c00755

**Published:** 2024-02-05

**Authors:** Yosta de Stigter, Harmen J. van der Veer, Bas J. H. M. Rosier, Maarten Merkx

**Affiliations:** † Laboratory of Chemical Biology, Department of Biomedical Engineering, Eindhoven University of Technology, 5600 MB Eindhoven, The Netherlands; ‡ Institute for Complex Molecular Systems, Eindhoven University of Technology, 5600 MB Eindhoven, The Netherlands

## Abstract

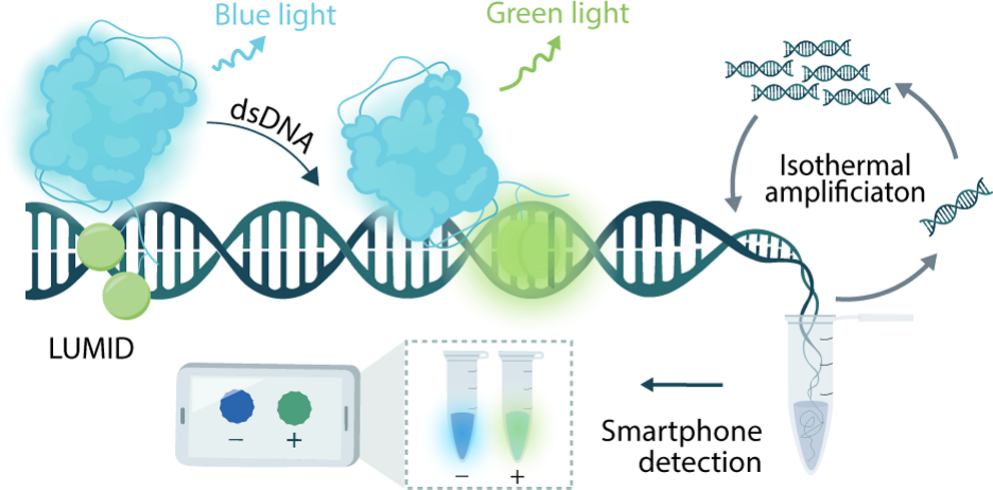

Rapid and sensitive DNA detection methods that can be conducted at the point of need may aid in disease diagnosis and monitoring. However, translation of current assays has proven challenging, as they typically require specialized equipment or probe-specific modifications for every new target DNA. Here, we present **Lu**minescent **M**ultivalent **I**ntercalating **D**ye (LUMID), off-the-shelf bioluminescent sensors consisting of intercalating dyes conjugated to a NanoLuc luciferase, which allow for nonspecific detection of double-stranded DNA through a blue-to-green color change. Through the incorporation of multiple, tandem-arranged dyes separated by positively charged linkers, DNA-binding affinities were improved by over 2 orders of magnitude, detecting nanomolar DNA concentrations with an 8-fold change in green/blue ratio. We show that LUMID is easily combined with loop-mediated isothermal amplification (LAMP), enabling sequence-specific detection of viral DNA with attomolar sensitivity and a smartphone-based readout. With LUMID, we have thus developed a tool for simple and sensitive DNA detection that is particularly attractive for point-of-need applications.

## Introduction

Sensitive detection of nucleic acids is key to a wide array of applications, ranging from infectious disease diagnosis and oncology to food and environmental safety monitoring and forensic DNA fingerprinting.^1−5^ Polymerase chain reaction (PCR)-based assays set the benchmark for nucleic acid detection, owing to their ability to amplify as few as 1–10 copies of target DNA to detectable concentrations.^6^ To facilitate quantification of target DNA, quantitative PCR (qPCR) allows for real-time detection utilizing fluorescent intercalating dyes that exhibit a strong increase in fluorescence upon binding nonspecifically to double-stranded DNA (dsDNA).^7^ Although qPCR is an established and powerful tool for sensitive DNA detection, the required setup can be prohibitive in situations where the use of expensive, specialized equipment (containing lasers, optics, and a thermal cycler) is limited or undesirable, for example, in point-of-need diagnostic testing.^8−10^

Isothermal nucleic acid amplification techniques have emerged as a promising alternative in which amplification is achieved at a constant temperature, obviating the need for thermocycling equipment.^11^ Loop-mediated isothermal amplification (LAMP) and recombinase polymerase amplification (RPA) are among the most widely used methods and have been combined with various sensing mechanisms suitable for point-of-care applications. Two examples that have received attention are the SHERLOCK and DETECTR platforms, which combine RPA amplification with highly specific DNA detection based on CRISPR-associated (Cas) proteins that cleave fluorescent reporter molecules upon binding the target nucleic acid.^12−14^ To circumvent a fluorescent readout, sensors based on bioluminescence provide a promising alternative. By employing luciferase enzymes that produce light without external excitation, it is possible to measure directly in complex matrices and without the use of specialized optical instrumentation, enabling simple detection through a smartphone camera. Several bioluminescent nucleic acid sensors have been developed, including bioluminescence resonance energy transfer (BRET)-based molecular beacons, a semisynthetic Luciferase-based Logic Device, and a recombinant zinc finger-luciferase protein in combination with uncoupled intercalating dyes.^15−17^ To further increase assay sensitivity, bioluminescent sensors have also been combined with isothermal nucleic acid amplification, for example, in work on split luciferase-DNA chimeras and the RPA-LUNAS assay.^18,19^

In contrast to the commercially available DNA dyes used in qPCR, most of the current nucleic acid sensors are sequence-specific and thus require biochemical modifications for every new target DNA. Although nonspecific probes such as pH-dependent colorimetric dyes and turbidity changes caused by magnesium pyrophosphate precipitate have successfully been combined with LAMP amplification,^20−22^ these do not directly detect DNA and are sensitive to matrix-dependent properties like pH.^16−18^ To our knowledge, only one nonspecific bioluminescent readout has been realized with the Bioluminescent Assay in Real Time (BART), but this method suffers from autoinhibition, which makes the output signal time-dependent and difficult to interpret and therefore less attractive for point-of-need testing.^23,24^

**Figure 1:**
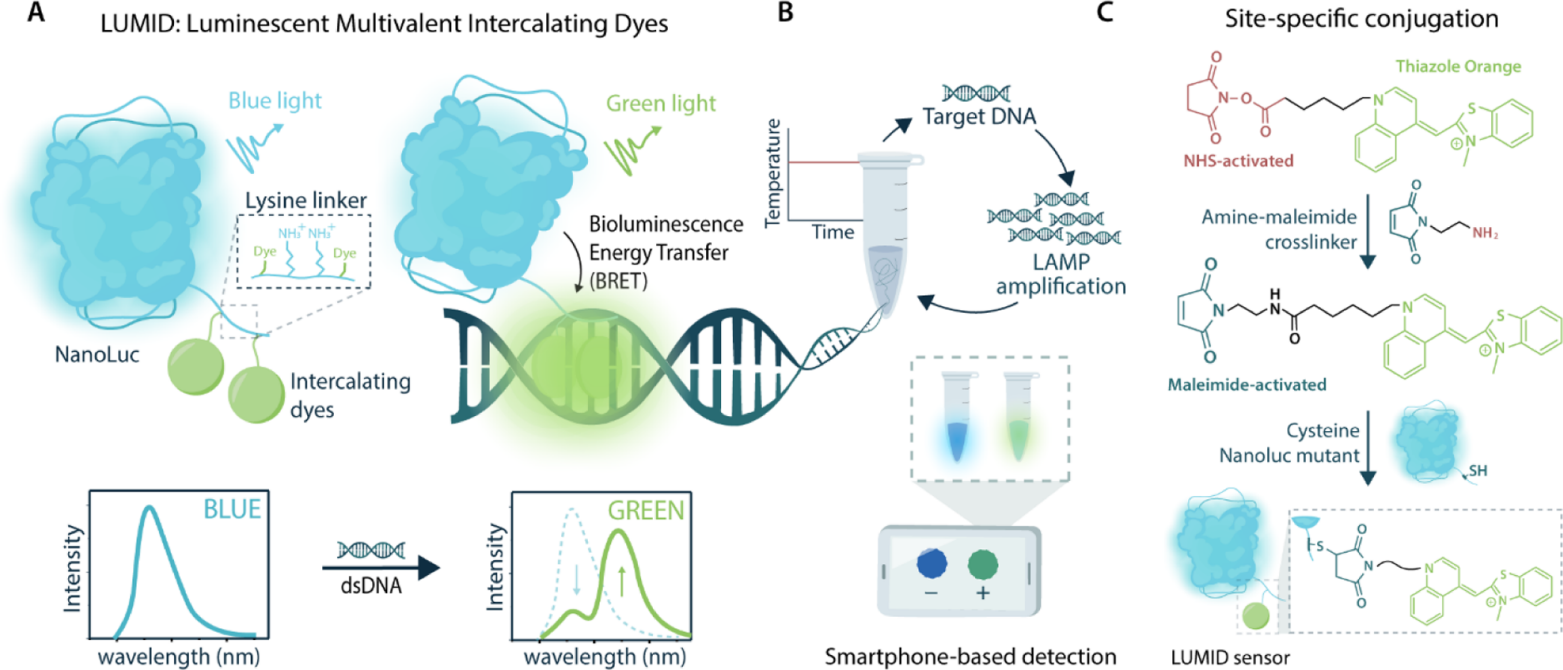
General overview of the LUMID sensor platform. (A) Schematic of the LUMID sensors. The probe consists of NanoLuc luciferase (blue) conjugated to intercalating dyes (green) that are separated by a positively charged lysine linker. In the absence of dsDNA, the intercalating dyes will be minimally fluorescent and the blue emission of NanoLuc can be observed (left). In the presence of dsDNA, the intercalating dyes bind to the dsDNA and allow for BRET, leading to green emission (right). (B) Final assay format. The LUMID sensors can be combined with LAMP to facilitate a preamplification step that introduces specificity and improves sensitivity. The results of such an assay can be read out using a smartphone camera. (C) Intercalating dye coupling procedure. The NHS-activated form of the intercalating dye thiazole orange is first coupled to a heterobifunctional 1-(2-aminoethyl)maleimide cross-linker to synthesize a maleimide-activated intercalating dye. Then, the maleimide-activated dye is coupled via thiol-maleimide chemistry to cysteines in NanoLuc to form the final LUMID sensor.

In this study, we introduce **Lu**minescent **M**ultivalent **I**ntercalating **D**yes (LUMIDs), generic bioluminescent sensor proteins that combine rapid, nonspecific detection of dsDNA with a simple camera-based readout that is suitable for point-of-care diagnostics. To this end, we developed ratiometric probes based on bioluminescence resonance energy transfer (BRET), consisting of one or more fluorescent intercalating dyes conjugated to the blue-light-emitting NanoLuc luciferase. Upon binding to dsDNA, the intercalating dyes function as BRET acceptors, leading to the transfer of energy from the luciferase to the dyes. This results in a robust and easy-to-detect bioluminescent color change from blue to green (Figure 1A). We demonstrate that multivalent dye interactions in combination with positively charged lysine linkers are essential for strong dsDNA binding and that BRET efficiency can be enhanced by locating the dyes closer to the active site of NanoLuc. Furthermore, we show that LUMID can be easily combined with a preamplification step using LAMP, enabling highly sensitive and specific detection of dsDNA with a smartphone-based readout (Figure 1B).

## Results and Discussion

### Single-Dye LUMID Variants

LUMID comprises the NanoLuc luciferase and one or more copies of the intercalating dye Thiazole Orange (TO), whose fluorescence strongly increases (>3000-fold) upon complexation with dsDNA to enable a dsDNA-dependent energy transfer from NanoLuc to the dye.^25^ To generate an efficient BRET-sensor, both a large spectral overlap and a short distance between the donor and acceptor molecules are essential, requiring a level of control over the position of the dye within NanoLuc. We therefore opted for a conjugation strategy using cysteine/maleimide chemistry in which different coupling sites within NanoLuc can be generated by incorporating cysteines through site-directed mutagenesis. Thiazole Orange was found particularly suitable, having appropriate spectral overlap with NanoLuc (emission max Nanoluc = 460 nm, excitation max TO = 514 nm) and being commercially available in an NHS-activated form that can be converted into a maleimide functionality with the use of an additional amine-maleimide cross-linker (Figure 1C).

**Figure 2:**
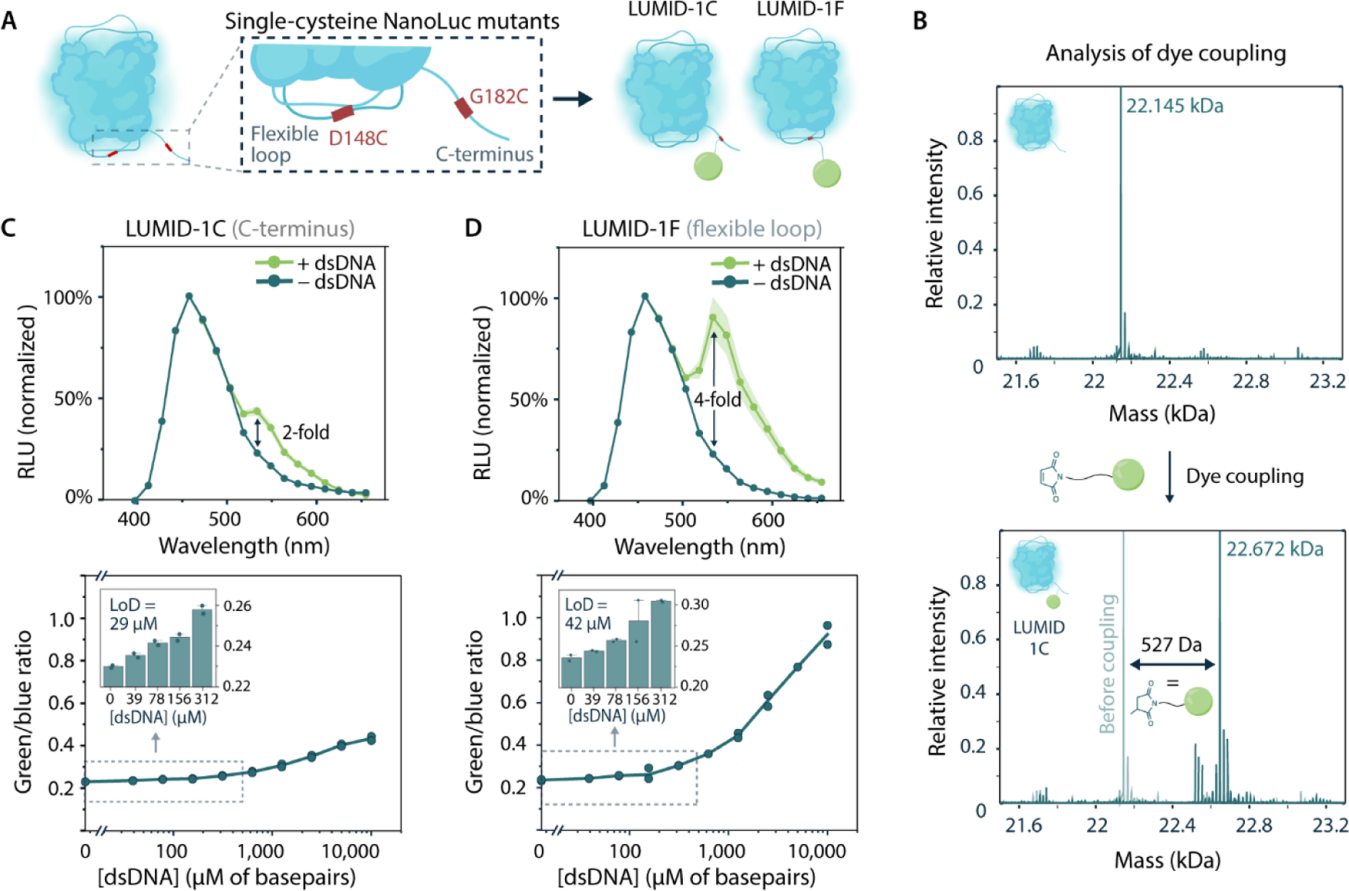
Synthesis and characterization of single-dye LUMID variants. (A) Schematic of conjugation positions within NanoLuc. Single-cysteine mutations (red) were incorporated either at the C-terminus (G182C) or in one of the flexible loop regions (D148C) of NanoLuc, which are coupled to the intercalating dye thiazole orange (TO) to create the LUMID-1C and LUMID-1F sensor, respectively. (B) Analysis of the dye coupling of the LUMID-1C protein. Mass spectra of LUMID-1C before (top) and after (bottom) the coupling of thiazole orange were obtained with Q-ToF LC-MS. In addition to the main peak with the expected molecular weight, minor additional peaks were observed that we attribute to hydrolysis of the maleimide (+18) and oxidation products (+16). (C, D) Bioluminescence titration with dsDNA of LUMID-1C (C) and LUMID-1F (D). The conjugates (1 nM) were added to sheared salmon sperm dsDNA ranging from 39.1 μM – 10 mM (in base pairs of DNA). Full emission spectra are displayed in the absence of dsDNA (blue) and in the presence of dsDNA (10 mM, green). The fold increase in the green/blue ratio is indicated in each graph. Insets represent the sensor response at low dsDNA concentrations, which allowed calculation of the limit of detection through linear regression, as indicated in the graphs. In panels (C, D), full- emission spectra (top) are represented as mean ± sd, and for the sensor response curves (bottom), individual data points are represented as circles and solid lines connect mean values. Data represent technical replicates, with *n* = 2 independent preparations of the dsDNA.

To establish proof of principle, we first synthesized single-dye LUMID variants by coupling single-cysteine NanoLuc variants through a heterobifunctional 1-(2-aminoethyl)maleimide cross-linker to the NHS-activated TO. The native cysteine in NanoLuc was mutated to a serine (C166S), and subsequently, single-cysteine mutations were incorporated at the C-terminus (G182C) and a flexible loop region (D148C) of NanoLuc (Figures 2A and S16). The C-terminal position is known to be suitable for conjugation without compromising on NanoLuc’s catalytic activity,^16^ whereas the position in the flexible loop is chosen based on its proximity to the active site. Both NanoLuc mutants were expressed in *Escherichia coli* and obtained in high yields after nickel affinity chromatography purification (∼100 mg/L, Figure S5). Next, the 1-(2-aminoethyl)maleimide cross-linker was conjugated to the NHS-activated TO (TO-NHS, Figure S7). Coupling reactions were performed on a 100-μL scale in DMSO by incubating 20 mM amino cross-linker with a 1:1 mol equiv of TO-NHS, overnight at RT. We hypothesized that a small excess of the NHS reactant should drive the reaction toward completion, consuming all free cross-linkers that could potentially interfere with conjugation to the protein. This way, an additional intermediate purification step could be avoided. Subsequent maleimide coupling reactions were performed on a 1 mL scale by incubating 10 μM of NanoLuc mutant with 10 mol equiv of maleimide-activated TO for 2 h at RT, covalently linking the intercalating dye to the cysteine residues. Quadrupole time-of-flight (Q-ToF) liquid chromatography–mass spectrometry (LC-MS) analysis showed complete labeling of the cysteine and no remaining nonconjugated protein (Figures 2B and S8).

In order to test the analytical performance of the single-dye LUMID sensors, bioluminescence titrations with dsDNA were performed (Figure 2C,D). Increasing concentrations of sheared salmon sperm dsDNA were added to 1 nM of sensor protein and incubated for 30 min at RT, followed by the addition of NanoLuc substrate. The construct with the dye positioned at the C-terminus (LUMID-1C) displayed a small increase in green light upon the addition of dsDNA, corresponding to a maximal 1.8-fold change in green/blue ratio with an apparent affinity in the low-mM range (*K*_D,app_ = 3.1 ± 0.3 mM, Figure S14) and a limit of detection of 29 μM (Figure 2C). Please note that concentrations are expressed in base pairs to reflect the nonsequence-specific mode of binding of LUMIDs to dsDNA. For example, an apparent *K*_D_ of 3 mM of base pairs corresponds to a molecular concentration of 2 μM for salmon sperm dsDNA with an average length of 1800 bp. Positioning the dye in the flexible loop region (LUMID-1F) yielded a similar affinity and limit of detection (*K*_D,app_ = 4.1 ± 0.7 mM and LOD = 42 μM, Figure S14), but with a larger, 3.8-fold maximal increase in green/blue ratio (Figure 2D). Since BRET is strongly distance-dependent, this difference in emission ratio can be attributed to the distance between the dye and the active site of NanoLuc in both mutants, i.e., positioning of the dye at the C-terminus leads to a larger distance to the active site compared to the flexible loop and therefore a reduced BRET efficiency. The *K*_D,app_ of nonconjugated TO was determined to be 139 ± 34 μM of base pairs, showing that protein conjugation attenuates dsDNA binding 20-fold (Figure S13). Although these initial LUMID sensors display low intrinsic affinities, the results validate our concept, demonstrating the DNA dependency of both sensor proteins expressed by an increase in the green/blue emission ratio that can be optimized through the positioning of the dyes.

### Multiple-Dye LUMID Variants

**Figure 3:**
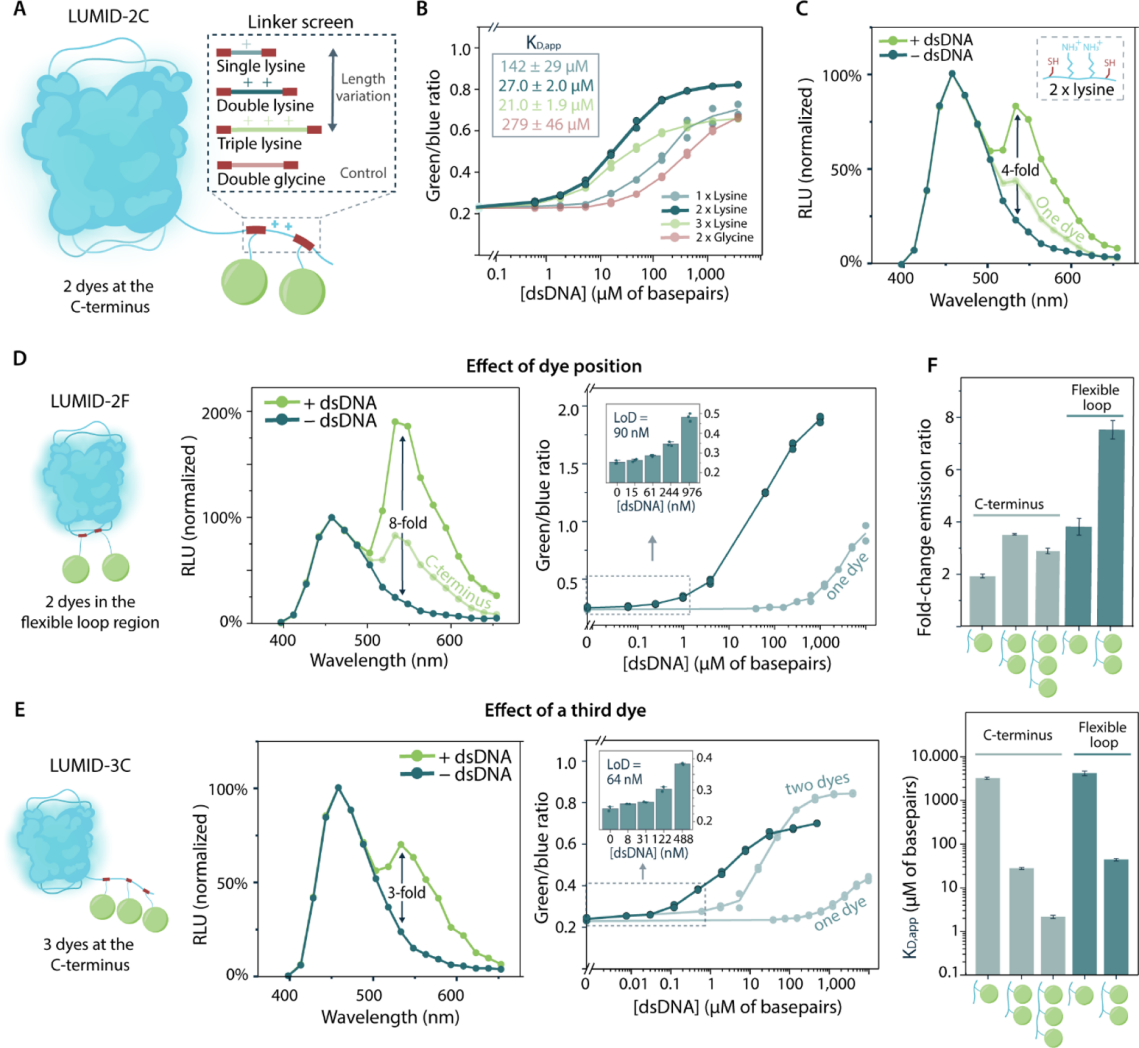
Optimization of LUMID through multivalent dye interactions. (A) LUMID sensor consisting of two dyes attached to the C-terminus was separated by different linkers, containing either one (gray), two (blue), or three (green) lysine residues or two glycine (red) residues as a negative control. (B–E) Bioluminescence titrations with dsDNA of the different sensor variants. Unless stated otherwise, experiments were performed in technical replicates with *n* = 2 independent preparations of the dsDNA, with 1 nM sensor protein. (B) Sensor response curves of the LUMID-2C sensor containing single- (gray), double- (blue), and triple-lysine (green) and double-glycine (red) linkers in the presence of various concentrations of salmon sperm dsDNA. (C) Full emission spectra of the LUMID-2C sensor with the double-lysine linker in the absence of dsDNA (blue) and in the presence of dsDNA (5000 μM, green). (D, E) Full emission spectra and sensor response curves of NanoLuc containing two dyes in the flexible loop ((D), LUMID-2F) and three dyes at the C-terminus ((E), LUMID-3C), both separated by a double-lysine linker. Insets represent the sensor response at low dsDNA concentrations, which allowed the calculation of the limit of detection through linear regression. The sensor response was measured in the presence of salmon sperm dsDNA ranging from 0.015 to 1000 μM of base pairs for LUMID-2F and 7.6 nM – 500 μM of base pairs for LUMID-3C. Full emission spectra are displayed in the absence of dsDNA (blue) and in the presence of dsDNA (1000 μM for LUMID-2F and 5000 μM for LUMID-3C, green). For LUMID-2F, experiments were performed in technical triplicates with *n* = 3 independent preparations of the dsDNA and the response curve was fitted with Hill coefficient *n* = 0.65. (F) Maximal change in emission ratio and affinity for dsDNA of the final LUMID-1C, LUMID-1F, LUMID-2C, LUMID-2F, and LUMID-3C variants. Bars represent average values ± standard deviation (s.d.).

After establishing the proof of principle for the LUMID concept, we sought to increase the affinity of the probes in order to reach concentrations relevant for point-of-need diagnostic applications. An attractive strategy to increase the overall affinity of the probes is to create a multivalent ligand consisting of a tandem of dyes that bind simultaneously to dsDNA. The development of a dimeric Thiazole Orange dye (TOTO) and small peptides containing two Acridine Orange dyes separated by two lysine residues were previously shown to increase the affinity to dsDNA by about two to three orders of magnitude compared to a monomeric variant.^26,27^ To apply the concept of multiple dyes with a positively charged linker to LUMID, we introduced a second intercalating dye in the proximity of the first intercalating dye at the C-terminus of NanoLuc, separated by lysine residues (LUMID-2C, Figure 3A). The amount of lysine residues was varied from one to three to find the optimal distance for intercalation of both dyes into the dsDNA, while glycine residues were used as a control to evaluate the effect of the positive charges on dsDNA binding. NanoLuc mutants were constructed through genetic insertion of the linker residues and a cysteine residue downstream of the cysteine already present at the C-terminus of the LUMID-1C variant (G182C) and expressed and purified in a similar procedure as the single-cysteine variants (∼100 mg/L, Figure S5). Next, coupling reactions with TO were performed using similar conditions as described before, and through Q-ToF LC-MS analysis, we observed complete TO labeling for all variants (Figures S9 and S10). A similar peak pattern was observed as for the single-dye variants.

To evaluate the binding properties of the different linker variants, bioluminescence spectra were obtained in the presence of increasing concentrations of sheared salmon sperm dsDNA. Using 1 nM of sensor protein and a 30 min incubation time before the addition of substrate, similar binding affinities were observed for the double-lysine linker (*K*_D,app_ = 27.0 ± 2.0 μM) and triple-lysine linker (*K*_D,app_ = 21.0 ± 1.9 μM), whereas the single-lysine linker displayed the weakest binding (*K*_D,app_ = 142 ± 29 μM, Figures 3B and S14). Furthermore, the double-lysine variant resulted in a 3.5-fold maximal increase in the green/blue ratio (Figure 3C), which was slightly higher than the 3.0-fold and 2.9-fold observed for the single- and triple-lysine variants. Control experiments with a double-glycine linker showed that replacing lysines with glycine residues results in a 10-fold decrease in affinity (*K*_D,app_ = 279 ± 46 μM, Figures 3B and S14), indicating that the positive charges contribute to enhanced dsDNA binding. Based on this screening, the double-lysine linker proved to be the most optimal for effective intercalating of both dyes, yielding a sensor with an over 100-fold increase in affinity and a 2-fold increase in dynamic range compared to the C-terminal single-dye variant.

To further explore the use of multivalent dye interactions to increase the sensor’s affinity and dynamic range, we introduced a second dye in the flexible loop region (LUMID-2F) and a third dye at the C-terminus (LUMID-3C), both separated by the optimal double-lysine linker (Figure 3D,E, left). Molecular cloning, protein expression, and purification were performed using the same conditions as previously described for the other multiple-dye LUMID sensors (yield ∼20–100 mg/L, Figure S6). Although obtaining high-quality MS data was hampered by poor ionization and/or the presence of protein-bound salts, Q-ToF LC-MS analysis showed main peaks consistent with double and triple TO conjugations of LUMID-2F and LUMID-3C, respectively (Figure S11). Bioluminescence titrations of LUMID-2F and −3C with dsDNA were performed using the same reaction conditions as described for the previous variants. A maximal 8-fold increase in green/blue ratio was observed for the variant with two dyes in the flexible loop (Figure 3D, middle) with an affinity comparable to that of the C-terminal variant (*K*_D,app_*=* 43.0 ± 2.9 μM, Figure S14). In line with the results obtained for the single-dye LUMID sensors, this illustrates that BRET efficiency is strongly distance-dependent and that positioning of the intercalating dyes in the flexible loop increases the energy transfer. The addition of a third dye to the C-terminus (LUMID-3C) showed a further, 10-fold improvement in affinity (*K*_D,app_*=* 2.1 ± 0.2 μM, Figure S14) when compared with the variant with 2 dyes at the C-terminus (Figure 3F). Interestingly, a decrease in the slope of the binding curve and a smaller dynamic range (2.9-fold) was observed, which could be due to the presence of small amounts of single- and double-conjugated species that result in a mixture of different affinity binders and inefficient simultaneous intercalation of all three dyes. Overall, these results illustrate that multiple-dye tandems can be incorporated in a flexible loop region to increase BRET efficiency and that a triple-dye tandem functions to enhance the affinity of the probe.

### Combining LUMID with LAMP

DNA targets are typically thousands of base pairs long, suggesting that the sensors developed here are capable of nonspecifically detecting such fragments in the picomolar range. Since most diagnostic applications, such as viral nucleic acid detection, require sequence-specific detection and a further increase in sensitivity to attomolar concentrations,^28,29^ we next explored combining our bioluminescent probes with different isothermal amplification steps (see Supporting Note). Of the methods explored, LAMP (Figure S4) was found most suitable due to the high dsDNA yield with minimal nonspecific amplification, which is essential to avoid large background signals when using a nonspecific readout. Motivated by the need for fast and simple diagnostics during the recent COVID-19 pandemic,^30−33^ LAMP reactions were designed to target the complementary DNA (cDNA) sequence of the nucleocapsid (N)-gene of the SARS-CoV-2 virus, exploring the feasibility of the LUMID probes for a sensitive and specific test for viral detection that can be used at the point of need.

**Figure 4:**
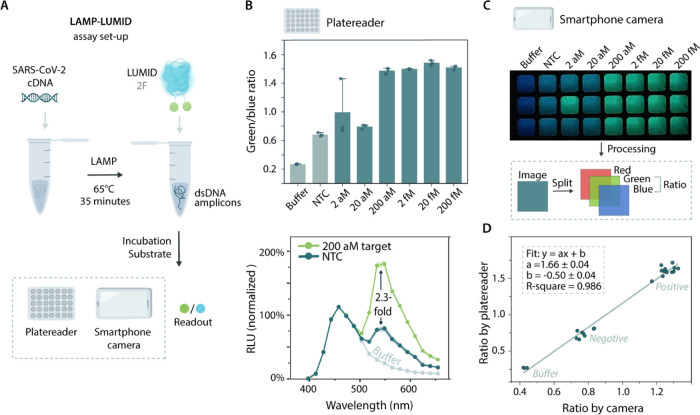
Development of a LAMP-LUMID assay for the detection of SARS-CoV-2 cDNA. (A) Schematic of the LAMP-LUMID assay setup. (B) Sensor response of LAMP-LUMID assay, using a plate reader for detection. Data represent technical replicates, with *n* = 3 independent preparations of the cDNA. Top: green/blue ratio with different initial concentrations of cDNA in the LAMP reaction, ranging from 2 aM (1.2 copies/μL) – 200 fM (120.000 copies/μL). Green/blue ratio was calculated by dividing the bioluminescent emission at 533 nm by emission at 458 nm and represented as mean ± standard deviation with circles depicting individual data points. Bottom: full emission spectra displayed in buffer (gray), LAMP reaction without target cDNA (NTC, blue), and LAMP reaction with 200 aM of target cDNA (green). RLU (relative luminescence units) was normalized to the NanoLuc peak at 458 nm, and the spectra (bottom) are represented as the mean ± standard deviation. (C) Sensor response of LAMP-LUMID, using a smartphone (Xiaomi mi 9 lite) inside a dark Styrofoam box. The photograph shows the sensor output for different input concentrations of cDNA, ranging from 2 aM (1.2 copies/μL) – 200 fM (120.000 copies/μL). Each column represents technical replicates, with *n* = 3 independent preparations of the cDNA. The same samples were used for detection with a smartphone camera (right) and the plate reader (left). (D) Correlation between the green/blue ratio calculated from the plate reader and smartphone camera for every sample tested in parts (B, C).

We developed an assay format in which serially diluted SARS-CoV-2 cDNA was amplified by using LAMP and subsequently combined with the LUMID-2F sensor for bioluminescent detection (Figure 4A). To this end, LAMP reactions were performed according to manufacturer’s instructions for 35 min at 65 °C, and then 1:1 (v/v) combined with 2 nM of sensor protein. Following the addition of substrate, DNA concentrations down to 200 aM (120 copies/μL) could be discriminated from the nontemplate control (NTC) with a 2.3-fold change in green/blue emission ratio (Figure 4B). To illustrate the potential for applications at the point of care, we also recorded the signal using a standard smartphone camera (Xiaomi mi 9 lite) in a dark Styrofoam box. Photographs taken of the same samples as used for the plate reader measurements showed a clear visual color change from blue to green for all DNA concentrations down to 200 aM (Figure 4C). The exact emission ratios were calculated from the blue- and green-color channels of the RGB image of the smartphone and showed a linear correlation with the ratio obtained from the plate reader measurements (Figure 4D). This reveals that although the absolute changes in emission ratio are different, the performance of the smartphone camera is comparable to that of the plate reader. For both detection methods, the amount of green light in the NTC was found to be higher than that in the control with only buffer, which can be ascribed to the long (∼40 bp) primers used in the LAMP reaction that form detectable secondary dsDNA structures without amplification. Although this primer-related background signal decreases the assay’s dynamic range, the observed change in emission ratio is robust and comparable to BRET-based sensors in general.^34^ With these results, we illustrate that our LUMID sensors can be combined with LAMP to create a specific and sensitive nucleic acid assay platform in which results can be obtained in approximately 1 h using simple, smartphone-based detection.

## Conclusions

In this study, we have introduced luciferase-intercalating dye conjugates as attractive bioluminescent alternatives for the well-known fluorescent intercalating dyes. The intercalating dyes present in LUMID both provide for high affinity, sequence-independent dsDNA binding and allow robust ratiometric bioluminescent detection, switching from blue to green emission in the presence of dsDNA. The use of multiple tandem-arranged dyes, positioned close to the active site and spaced by double-lysine linkers, was a requisite for improved sensor performance, increasing the sensor affinities to the low-μM range (2.1–43 μM of base pairs) and the dynamic range to an 8-fold maximal increase in green/blue ratio. The best-performing LUMID-2F variant was successfully combined with a LAMP preamplification step into an assay for the complementary DNA of the nucleocapsid-gene of the SARS-CoV-2 virus, with attomolar sensitivity and smartphone-based readout.

We showed that by incorporating double- and triple-dye tandems, sensor affinities could be improved by a factor of 10^2^ to 10^3^. Although higher-affinity sensing is not always required or optimal, for instance, in our LAMP-LUMID assay that involves an additional amplification step and is sensitive to background binding to primers, probes with a further increase in affinity might be beneficial for direct sensing applications. To obtain such probes, optimization of conjugation conditions and additional purification steps to remove incomplete coupling products would be required. Furthermore, the sensor’s dynamic range may be further improved by screening additional dye positions for BRET efficiency and dsDNA binding, in particular those in the loop regions flanking the active site of NanoLuc.

The combination of LUMID with LAMP-based isothermal amplification provides an attractive tool for point-of-need nucleic acid diagnostics. In contrast to commonly used fluorescent probes, our bioluminescent LUMID sensors do not require external excitation, which makes them compatible with a simple smartphone-based readout. The generic nature makes the sensors well-suited for rapid, off-the-shelve use, obviating the need for chemical synthesis of a probe for every new target DNA. While the assay time in the current, 2-step setup could likely be decreased by using shorter incubation times, we also aim to increase the thermostability of the LUMID system to enable rapid, real-time monitoring in a one-pot assay with LAMP.^35^ For future applications, we envision the integration of the LAMP-LUMID combination into cheap paper-based devices or microfluidic chips,^36−38^ creating a simple and sensitive diagnostic tool that is user-friendly and particularly suitable for application in a low-resource setting. Finally, LUMID might also find use in other applications that currently rely on fluorescent intercalating dyes, such as dsDNA detection in imaging and dsDNA quantification.

## Methods

### Cloning

The pET28a vector containing DNA encoding the NanoLuc luciferase with an N-terminal Strep-tag and C-terminal hexahistidine-tag was ordered from GenScript. Site-directed mutagenesis to mutate the native cysteine to a serine (C166S) and introduce new cysteine and lysine residues was carried out using the QuikChange Lightning Site-Directed Mutagenesis kit (Agilent) using specific primers according to the manufacturer’s instructions (Table S3). All cloning and mutagenesis results were confirmed by Sanger sequencing (BaseClear). Supporting Figure S16 and Table S1 show the DNA and corresponding amino acid sequences of NanoLuc with specific mutation sites.

### Protein Expression and Purification

The plasmids encoding NanoLuc were transformed into chemically competent *E. coli* BL21 (DE3) and cultured in 2YT medium (16 g of peptone, 5 g of NaCl, 10 g of yeast extract per liter) supplemented with 50 μg/mL kanamycin. At OD_600_ = 0.6, protein expression was induced using 1 mM isopropyl β-d-1-thiogalactopyramoside (IPTG) overnight at 20 °C. Subsequently, cells were harvested by centrifugation and lysed using Bugbuster protein extraction reagent (Novagen), supplemented with Benzonase endonuclease (Novagen). Proteins were purified using Ni^2+^-NTA affinity chromatography, after which the elution fractions were exchanged in storage buffer (100 mM Tris-HCl, 150 mM NaCl, pH 8.0). Sodium dodecyl sulfate–polyacrylamide gel electrophoresis (SDS–PAGE) was used to determine the protein purity (Figures S5 and S6). Correct protein mass was confirmed by Q-ToF LC-MS (WatersMassLynx v4.1) using MagTran v1.03 for MS deconvolution (Figures S8–S11). Mass spectra were obtained using a 1 μL injection volume, containing 0.1 mg mL^–1^ of protein in Q-ToF buffer (Milli-Q, 0.1% formic acid). Purified proteins were stored at −80 °C until conjugation.

### Conjugation of NHS-Activated Dye to 1-(2-Aminoethyl)maleimide Cross-Linker

An amine-reactive *N*-hydroxysuccinimide (NHS) ester of Thiazole Orange (TO) was obtained from Biotium (cat. no. 40073), and a 1,2-(aminoethyl)maleimide cross-linker was obtained from Sigma-Aldrich (cat. #809322). Both components were dissolved in DMSO and added into a 100 μL-reaction containing 24 mM (1 equiv) of TO, 20 mM (0.8 equiv) 1,2-(aminoethyl)maleimide cross-linker, and 72 mM of DIPEA (3 equiv). Reactions were incubated overnight at RT with continuous shaking at 450 rpm. The correct mass of the maleimide-conjugated TO was confirmed using LC-MS (Figure S7), and the unpurified reaction mixture containing maleimide-activated TO was stored at −30 °C until further use.

### Conjugation of Maleimide-Activated Dyes to NanoLuc

Before conjugating NanoLuc to the maleimide-activated TO, the protein was first reduced through incubation with 5 mM of TCEP for 1 h at RT with continuous shaking at 500 rpm and subsequently buffer exchanged to a sodium phosphate buffer (100 mM NaPO_4_, 25 μM TCEP, pH 7.0) using a PD-10 desalting column (GE Healthcare). Then, the maleimide-activated TO was added in a 10-fold molar excess (relative to the amount of cysteines) to 10 μM of reduced NanoLuc and allowed to react for 2 h at RT with continuous shaking at 500 rpm. The NanoLuc-dye conjugates were purified by a PD-10 desalting column to remove excess dye and simultaneously buffer exchanged to phosphate-buffered saline (PBS) (100 mM NaPi, 150 mM NaCl, pH 7.2). The coupling efficiency and correct mass of the NanoLuc-dye conjugates were confirmed by Q-ToF LC-MS (WatersMassLynx v4.1), using MagTran v1.03 for MS deconvolution (Figures S8–S11). Mass spectra were obtained using a 2 μL injection volume containing 0.1 mg mL^–1^ of protein in Q-ToF buffer (Milli-Q, 0.1% formic acid, 10% acetonitrile).

### Bioluminescent Titrations with dsDNA

Bioluminescent assays were performed at sensor protein concentrations of 1 nM in a total volume of 20 μL of PBS buffer (pH 7.4, 0.1% (w/v) BSA, 5% DMSO) in a PerkinElmer flat white 384-well Optiplate. Sheared Salmon Sperm dsDNA fragments of ∼2000 bp were ordered from Thermo Fisher and diluted to a concentration range of 0.015 μM – 10 mM, measured in terms of the number of base pairs. After incubation of sensor proteins with dsDNA fragments for 30 min at RT, NanoGlo substrate (Promega, N1110) was added at a final dilution of 1:1000. Luminescence spectra were recorded in a plate reader (Tecan Spark 10M) between 398 and 653 nm with a step size of 15 nm, a bandwidth of 25 nm, and an integration time of 100 ms. The green/blue ratio was calculated by dividing the bioluminescent emission at 533 nm by emission at 458 nm. Sensor response curves were fitted in Origin (2020) using a Hill function with offset to determine an apparent affinity using the equation below. The Hill coefficient was fixed to 1, except for the LUMID-3C. The model fits and fitting parameters of all titration curves can be found in Supporting Figure S14.
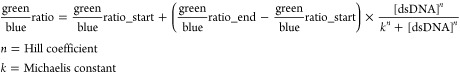
The limit of detection values was calculated in Origin (2020) through linear regression of the sensor response (green/blue ratio) over the dsDNA concentration for a limited range of concentrations in the linear regime. The LOD was then determined to be the concentration dsDNA at which the sensor response was equal to the sum of *y*-intercept and three times the standard deviation of *y*-intercept using the linear regression line.

### SARS-CoV-2 Assays Using LAMP

LAMP primers targeting the cDNA sequence of the Nucleocapsid (N)-gene of the SARS-CoV-2 virus were designed using the NEB LAMP Primer Design Tool and ordered from IDT. The sequence of the N-gene can be found in Figure S18, and the used primer set can be found in Table S6 (primer set 1). Primers were diluted to a 10× concentrated stock, containing 16 μM of inner primers, 2 μM of outer primers, and 4 μM of loop primers. A positive control plasmid containing SARS-CoV-2 cDNA sequences was provided by the Free Genes Project and using specific primers, the nucleocapsid (N)-gene sequence was PCR amplified from this plasmid. The N-gene target DNA was serially diluted to 25× concentrated stocks ranging from 50 aM – 5 pM. Positive LAMP reactions were assembled in a UV PCR cabinet by combining 1× isothermal amplification buffer (NEB), 6 mM of MgSO_4_ (NEB), 1.4 mM of dNTPs (NEB), 1× LAMP primer mix, and 1× target DNA in a total volume of 24 μL. For the nontemplate control, similar conditions were used, only interchanging the target DNA for Milli-Q water. Reactions were kept on ice during the assembly process. To initiate the reactions, Bst 2.0 polymerase (8 U, NEB) was added followed by incubation at 65 °C for 35 min. Next, 10 μL of each LAMP reaction was combined with 10 μL of LUMID-2F sensor (2 nM in PBS buffer (pH 7.4, 0.1% (w/v) BSA, 5% DMSO)) in a PerkinElmer flat white 384-well Optiplate. For a negative control, the LAMP reaction mixture was substituted with PBS buffer. After incubation for 30 min at RT, NanoGlo substrate (Promega, N1110) was added at a final dilution of 1:1000. Luminescence spectra were recorded in a plate reader (Tecan Spark 10M) between 398 and 653 nm with a step size of 15 nm, a bandwidth of 25 nm and an integration time of 100 ms. The green/blue ratio was calculated by dividing bioluminescent emission at 533 nm by emission at 458 nm. The luminescence signal was also recorded using a smartphone (Xiaomi mi 9 lite) camera through a hole in a Styrofoam box to exclude the surrounding light. Photographs were taken with an exposure time of 32 s and an ISO value of 3200. Resulting RAW images were converted to TIFF files, and mean blue (B) and green (G) intensities per well were extracted from split RGB channels in ImageJ (v1.53q). The linear correlation between camera and plate reader green/blue ratios was calculated in Origin (2020).

## Data Availability

The data that support the plots within this paper and other findings of this study are available from the corresponding author (m.merkx@tue.nl) upon reasonable request.

## Supporting Information Available

Additional screening of isothermal amplification methods; SDS–PAGE analysis of purified proteins; LC-MS spectra for all coupling reactions; absorbance and dsDNA titration experiments with thiazole orange; ssDNA titration experiments with LUMID-2F; model fits of DNA titrations; DNA and protein sequences (PDF)
