# Supplementary material for: Bioluminescent Intercalating Dyes for Ratiometric Nucleic Acid Detection
Source: ACS Chem Biol. 2024 Feb 5;19(2):575–83. doi: 10.1021/acschembio.3c00755 (PMC10877566; doi:10.1021/acschembio.3c00755)
Supplement: Supplementary file 1 — cb3c00755_si_001.pdf [file cb3c00755_si_001.pdf]

Supporting information

Bioluminescent Intercalating dyes for Ratiometric Nucleic Acid Detection.

Yosta de Stigter[1,2], Harmen J. van der Veer[1,2], Bas J. H. M. Rosier[1,2†], and Maarten Merkx[1,2*]

1. Laboratory of Chemical Biology, Department of Biomedical Engineering, Eindhoven University of Technology, 5600 MB Eindhoven, The Netherlands.
2. Institute for Complex Molecular Systems, Eindhoven University of Technology, 5600 MB Eindhoven, The Netherlands.

*Email: m.merkx@tue.nl

Table of Contents

Supplementary note | Screening of isothermal amplification methods **4**

Recombinase Polymerase Amplification 4

Figure S1. Effect of DMSO on non-specific amplification in RPA 4

Figure S2. Real-time RPA reactions for optimized primer sets 5

Figure S3. Sensor response to RPA reactions 6

Loop-mediated Isothermal Amplification 7

Figure S4. Real-time LAMP reactions 7

Supplementary Figures **8**

Figure S5. SDS-PAGE analysis of NL1C, NL1F, NL2C-1K, NL2C-2K, NL2C-3K and NL2C-2G purification 8

Figure S6. SDS-PAGE analysis of NL3C and NL2F purification 9

Figure S7. Overview of the amine-maleimide crosslinker coupling to the NHS-activated form of Thiazole Orange 10

Figure S8. ESI-QTOF mass spectra of LUMID-1C and LUMID-1F proteins, before and after dye coupling 11

Figure S9. | ESI-QTOF mass spectra of LUMID-2C with single- and double-lysine linker, before and after dye coupling 12

Figure S10. ESI-QTOF mass spectra of LUMID-2C with triple-lysine and double-glycine linker, before and after dye coupling 13

Figure S11. ESI-QTOF mass spectra of LUMID-3C and LUMID-2F proteins, before and after dye coupling 14

Supplementary Figure 12. Absorbance spectrum maleimide-activated Thiazole Orange 15

Supplementary Figure 13. Titrations of maleimide-activated Thiazole Orange with dsDNA 15

Supplementary Figure 14. Model fits of different LUMID variants. 16

Supplementary Figure 15. Titrations LUMID-2F with ssDNA and dsDNA 17

Supplementary Figure 16. DNA and amino acid sequence of NanoLuc 17

Supplementary Figure 17. Sequence target DNA of the SARS-CoV-2 ORF1a complementary DNA 18

Supplementary Figure 18. Sequence of the SARS-CoV-2 nucleocapsid gene complementary DNA (ORF_N) 18

Supplementary tables **19**

table S1. Mutation sites of the different NanoLuc variants 19

table S2. Calculated molecular weights for the different NanoLuc construct without dyes, with dyes and with hydrolyzed maleimide functionalities 19

table S3. Sequences of the primers used for mutagenesis 20

table S4. Sequences of the RPA primers targeting ORF_1a 20

table S5. Sequences of the RPA primers targeting ORF_N 21

table S6. Sequences of the LAMP primers targeting ORF_N 21

Supplementary references **22**

Supplementary note - ***Screening of isothermal amplification methods***

We explored the use of Recombinase Polymerase Amplification (RPA) and Loop-mediated Isothermal amplification (LAMP) as an initial pre-amplification step suitable to allow the application of LUMID for point-of-care applications. RPA and LAMP are among the most widely used isothermal amplification methods and can be performed at a constant temperature of 37-42°C and 60-65°C, respectively. Both methods were screened for non-specific amplification and dsDNA yield by following the reactions in real-time using the fluorescent intercalating dye EvaGreen, representative for the intercalating dyes used in our sensors.

Recombinase Polymerase Amplification

In the RPA reactions, all non-template controls showed a substantial amount of non-specific amplification (for instance, see supplementary Figure S1) that can be attributed to the presence of primer-dimers and primer-related secondary structures, which form a small piece of dsDNA that facilitates the binding of the polymerase enzyme and subsequent amplification. Since our sensors bind dsDNA non-specifically, the presence of such non-specific amplicons gives rise to high background signals. Although additives, such as DMSO, were reported to minimize this effect, we found that adding 5% DMSO to our reactions did not decrease the amount of non-specific amplification relative to the reactions that contained target DNA (supplementary Figure S1)[1]. New primers were designed to minimalize primer overlap, but the non-specific amplification seemed inherent to the RPA reaction as a result of the relatively low reaction temperatures (supplementary Figure S2). Furthermore, our highest-affinity LUMID sensors were only just capable of detecting the amount of dsDNA resulting from an RPA reaction (supplementary Figure S3), suggesting that the dsDNA yield is relatively low.

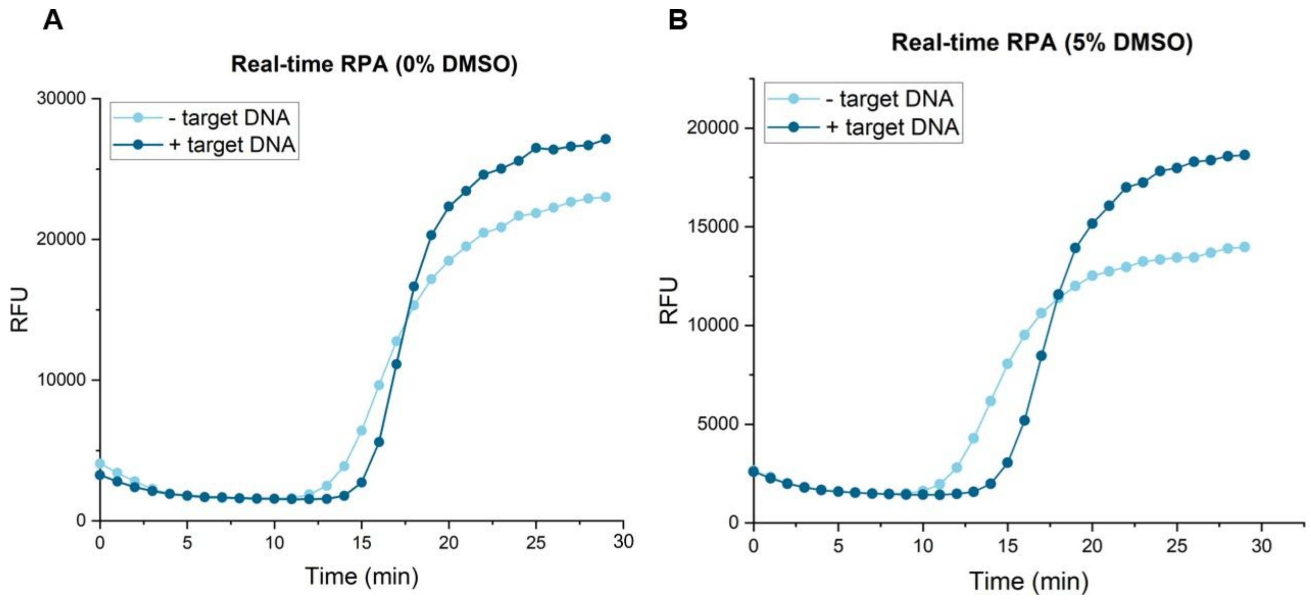

Figure S1 | Effect of DMSO on non-specific amplification in RPA. A RPA reactions were performed according to manufacturer's instructions with the addition of EvaGreen dye (1x), using 1000 copies/µL of SARS-CoV-ORF1a complementary DNA (+ target DNA) and specifically designed primers (see supplementary table 4). For the no template control (- target DNA), the target DNA was exchanged for water. Using an excitation wavelength of 488 nm, the fluorescence at 530 nm was measured every 30 seconds for 40 minutes at 39°C. **B** Similar conditions as described in A were used, but with the addition of 5% DMSO to the reaction mixture.

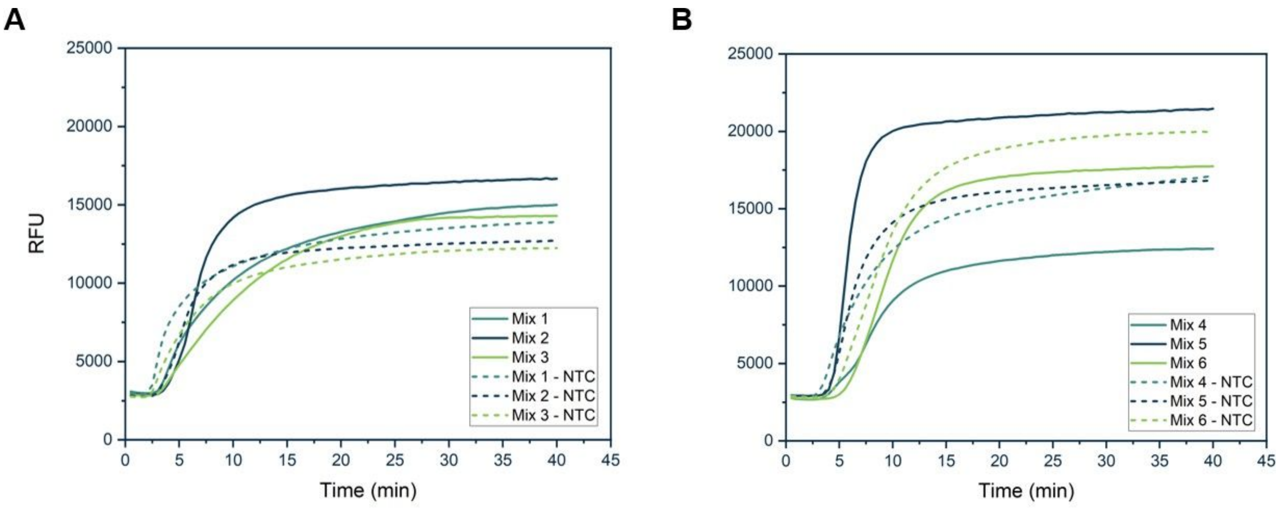

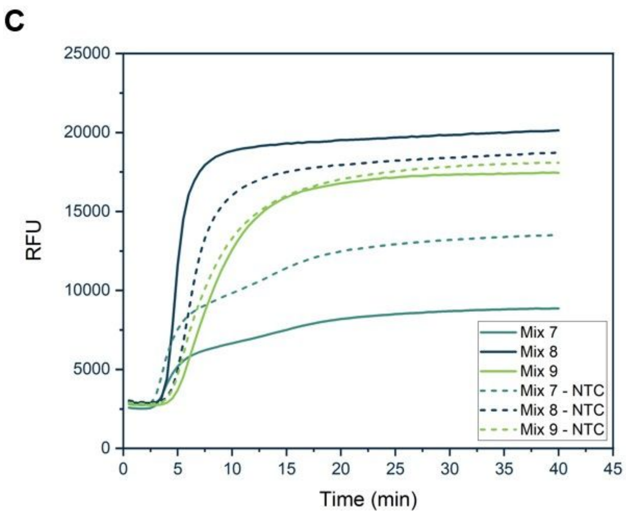

D

Mix	Primers
1	Forward 1 – Reverse 1
2	Forward 1 – Reverse 2
3	Forward 1 – Reverse 3
4	Forward 2 – Reverse 1
5	Forward 2 – Reverse 2
6	Forward 2 – Reverse 3
7	Forward 3 – Reverse 1
8	Forward 3 – Reverse 2
9	Forward 3 – Reverse 3

Figure S2 | Real-time RPA reactions for optimized primer sets. RPA reactions were performed according to manufacturer's instructions with the addition of EvaGreen dye (1x), using 1000 copies/µL of SARS-CoV-2 Nucleocapsid-gene complementary DNA and specifically designed primers (see supplementary table 5). For the no template control (NTC), the target DNA was exchanged for water. Fluorescence was monitored every 30 seconds for 40 minutes at 39°C, using SYBR mode (BioRad CFX Real-Time PCR Detection System). **A** Real-time curves for primer sets 1-3. **B** Real-time curves for primer sets 4-6. **C** Real-time curves for primer sets 7-9. **D** Overview of the primer combinations corresponding to the different mixes indicated in the graphs.

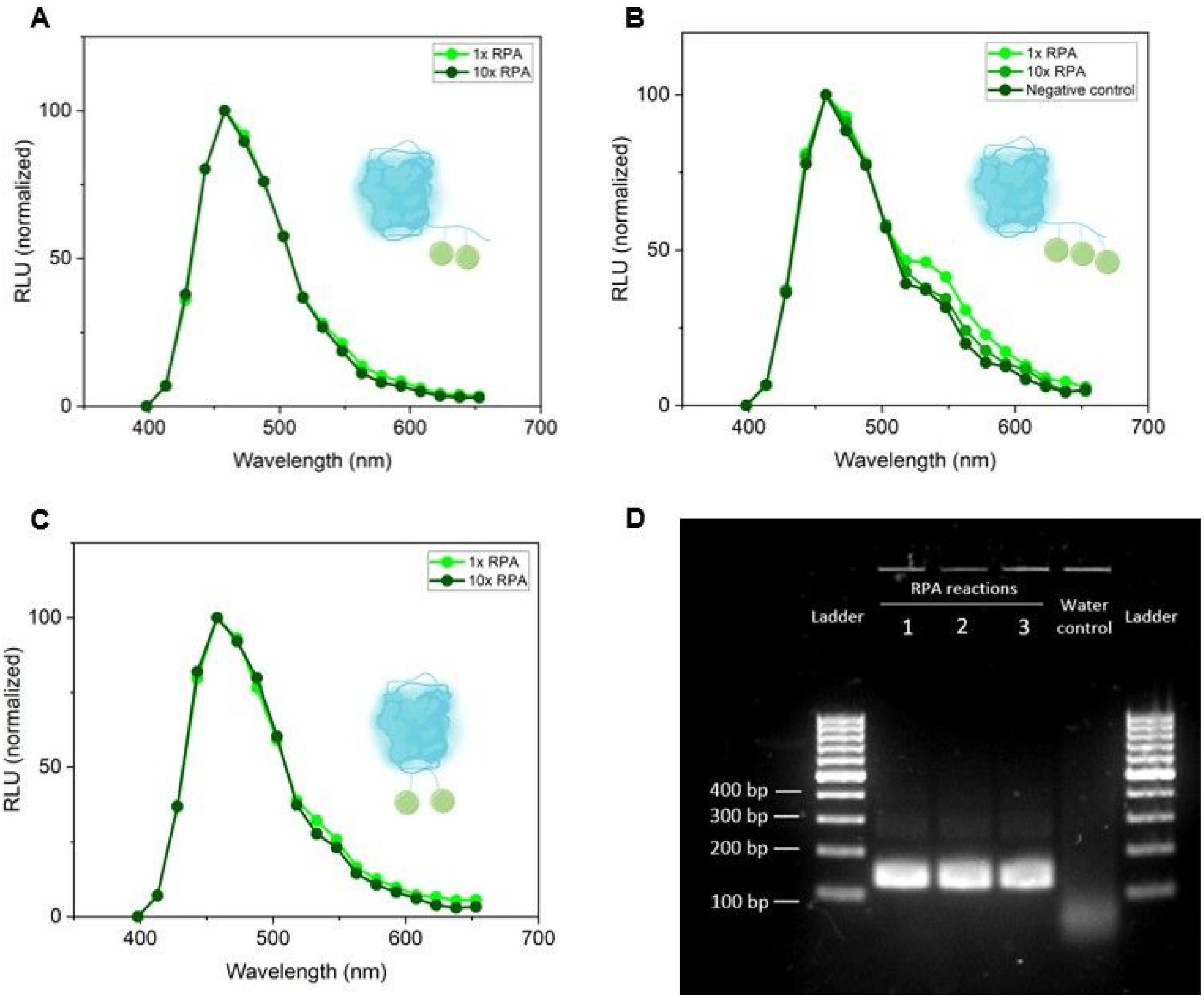

Figure S3 | Sensor response to RPA reactions. RPA reactions were performed according to manufacturer's instructions, using 1000 copies/µL of SARS-CoV-2 ORF1a complementary DNA and specifically designed primers (see supplementary table 4). For the negative control, the target DNA was exchanged for water. Reactions were incubated for 30 minutes at 39°C, and diluted 1:1 (v/v) with 2 nM of sensor (1xRPA), or 1:19 (v/v) with 1 nM of sensor (10xRPA). Following 30 minutes of incubation, NanoGlo substrate (1000x dilution) was added, after which the emission intensity in the range of 398-653 nm was measured. RLU (relative Luminescence units) was normalized to the NanoLuc peak at 458 nm. **A** NL2C sensor. **B** NL3C sensor. **C** NL2F sensor. **D** Gel analysis of 3 different RPA reactions using a 1.5% agarose gel stained with 1 x SYBR safe. Gel was run for 40 minutes at 120 V in 1xTAE. The DNA bands were visualized using 470-nm blue light.

Loop-Mediated Isothermal Amplification

In contrast to the RPA reactions, LAMP reactions did not show non-specific amplification in the non-template controls (supplementary Figure S4). Although we observed some background that can be ascribed to the long (~40 bp) primers that form detectable dsDNA structures at room temperature, this was effect was predictable and small compared to the positive reactions. The LAMP amplicons could be detected with a large increase in green/blue ratio to ~1.8, which indicates that the dsDNA yield in the LAMP reaction is higher than for the RPA reaction.

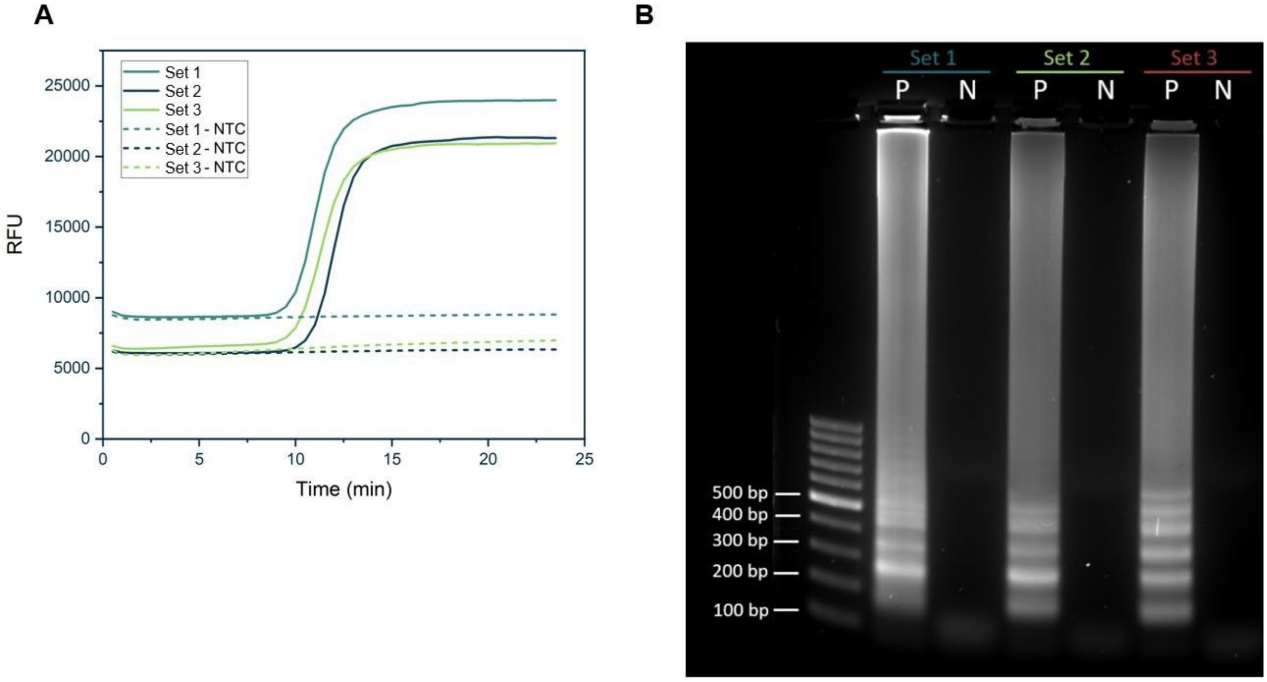

Figure S4 | Real-time LAMP reactions. LAMP reactions (Bst 2.0, NEB) were performed according to manufacturer's instructions with the addition of EvaGreen dye (1x), using 1000 copies/μL of SARS- CoV-2 Nucleocapsid-gene complementary DNA and specifically designed primers set (see supplementary table..). For the no template control (NTC), the target DNA was exchanged for water. Fluorescence was monitored every 30 seconds for 30 minutes at 65°C, using SYBR mode (BioRad CFX Real-Time PCR Detection System). **A** Real-time fluorescence curves for the different primer sets. **B** Gel analysis of LAMP reactions using different primer sets. LAMP reactions were loaded onto a 1.5% agarose gel stained with 1 x SYBR safe, and run for 40 minutes at 120 V in 1xTAE buffer.

Supplementary Figures

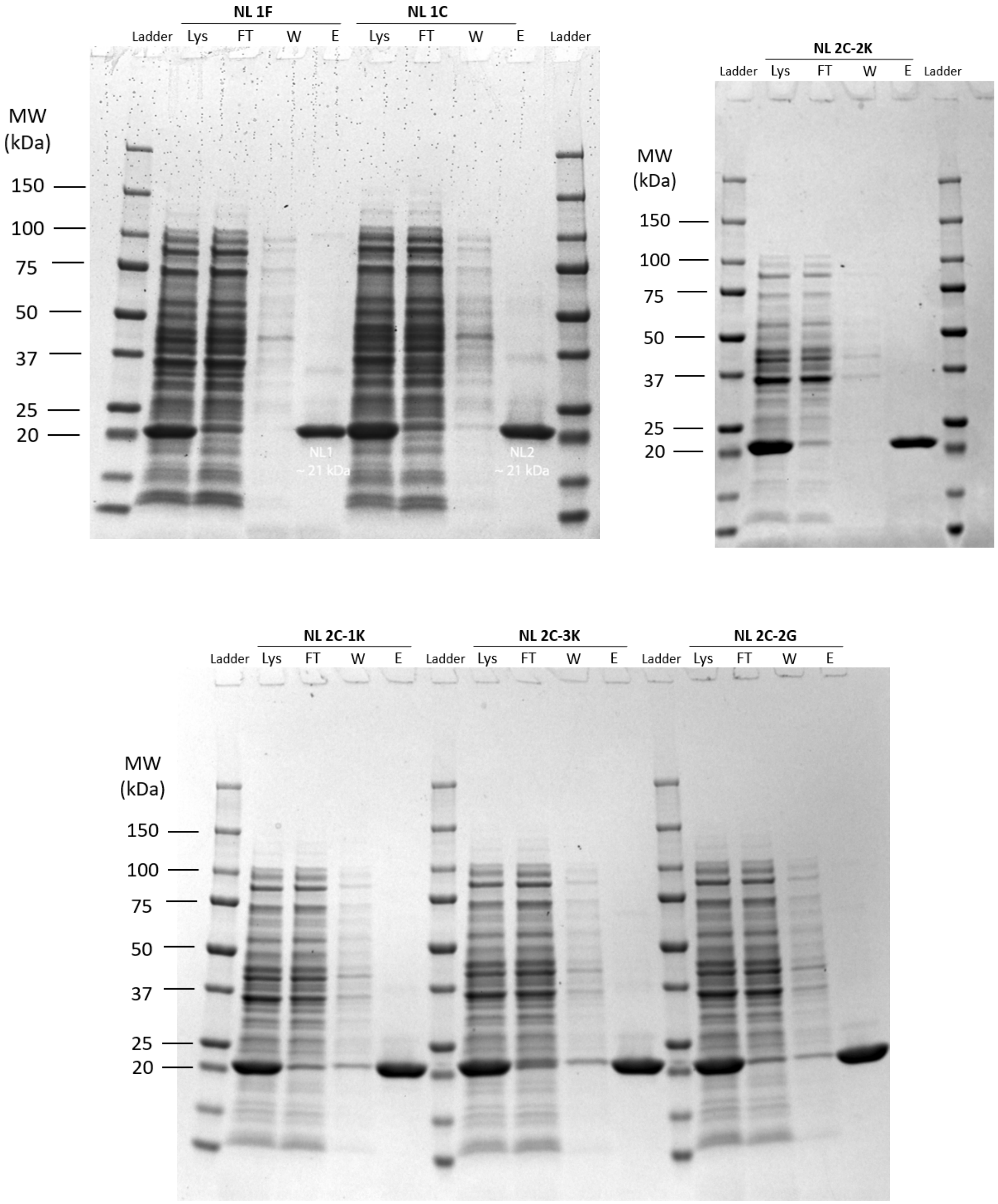

Figure S5 | SDS-PAGE analysis of NL1C, NL1F, NL2C-1K, NL2C-2K, NL2C-3K and NL2C- 2G purification. The NL1C, NL1F, NL2C-1K (single-lysine), NL2C-2K (double-lysine), NL2C-3K (triple-lysine) and NL2C-2G (double-glycine) proteins, used to generate corresponding LUMID variants, were expressed in *E. coli* and purified by using Ni^{2+} affinity chromatography. Lys: the supernatant of cell lysate; FT/W/E: Ni^{2+} affinity chromatography flow through, wash and elution samples. Marker: Precision Plus Protein™ marker (BioRad).

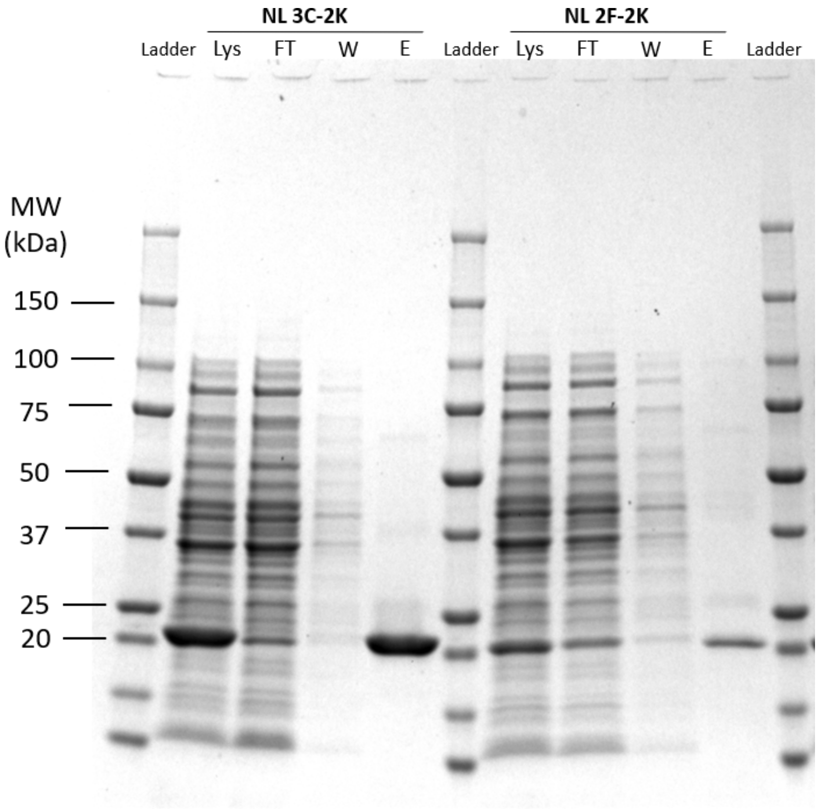

Figure S6 | SDS-PAGE analysis of NL3C and NL2F purification. The NL3C and NL2F proteins, used to develop corresponding LUMID variants, were expressed in *E. coli* and purified by using Ni^{2+} affinity chromatography. Lys: the supernatant of cell lysate; FT/W/E: Ni^{2+} affinity chromatography flow through, wash and elution samples. Marker: Precision Plus Protein™ marker (BioRad)

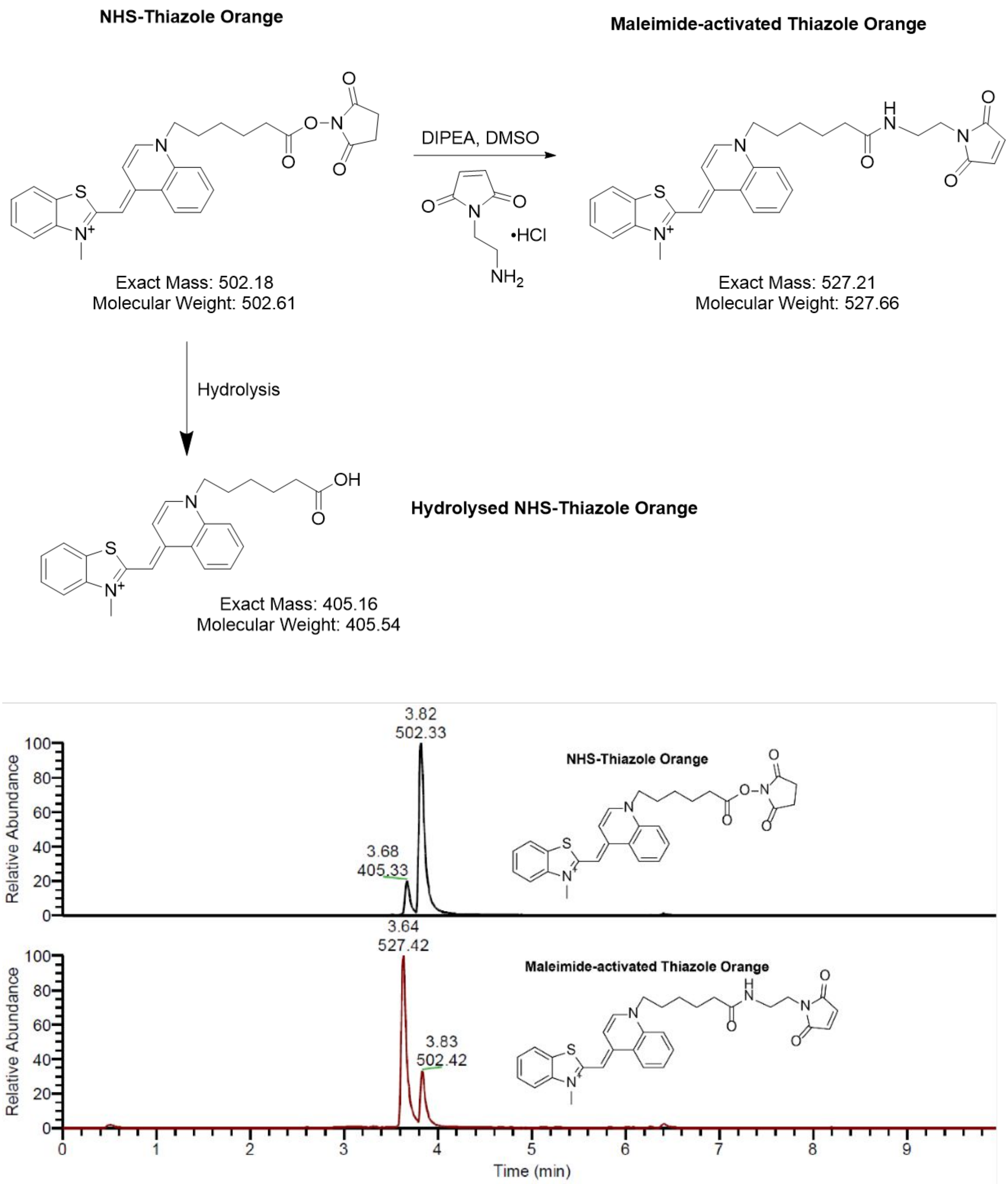

Figure S7 | Overview of the amine-maleimide crosslinker coupling to the NHS-activated form of Thiazole Orange. A Reaction scheme for coupling of the maleimide-activated crosslinker to NHS-TO. Molecular masses before and after coupling are indicated at the bottom of the structures. **B** LCMS results of NHS-TO before the coupling of the crosslinker (top) and after the coupling of the crosslinker (bottom). Molecular masses are indicated at the top of the chromatogram peaks.

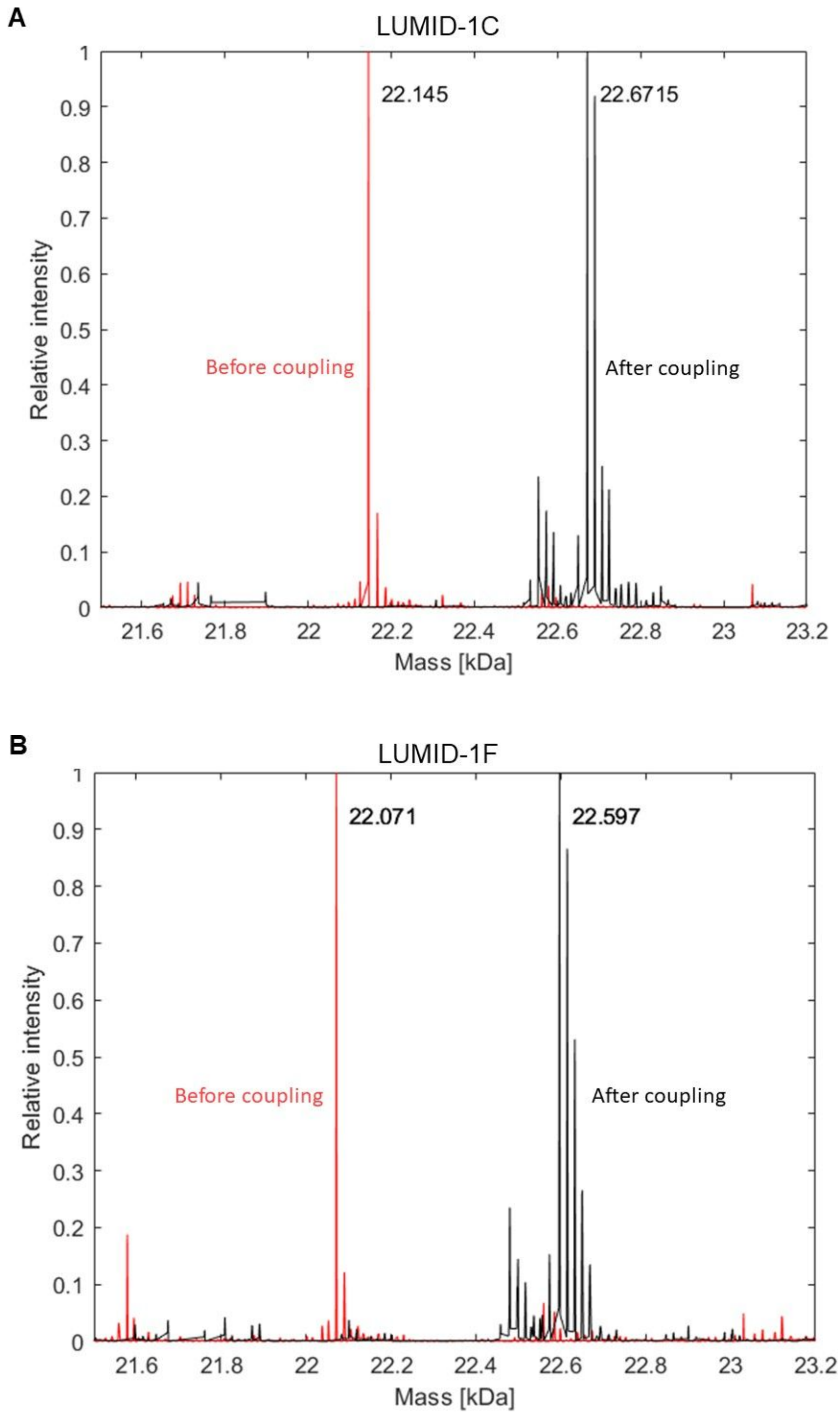

Figure S8 | ESI-QTOF mass spectra of LUMID-1C and LUMID-1F proteins, before and after dye coupling. A LUMID-1C (calculated mass without dye: 22.145 kDa, with dye: 22.672 kDa). **B** LUMID- 1F (calculated mass without dye: 22.071 kDa, with dye: 22.598 kDa, with dye). See supplementary table S2 for molecular weight calculations, including possible modifications.

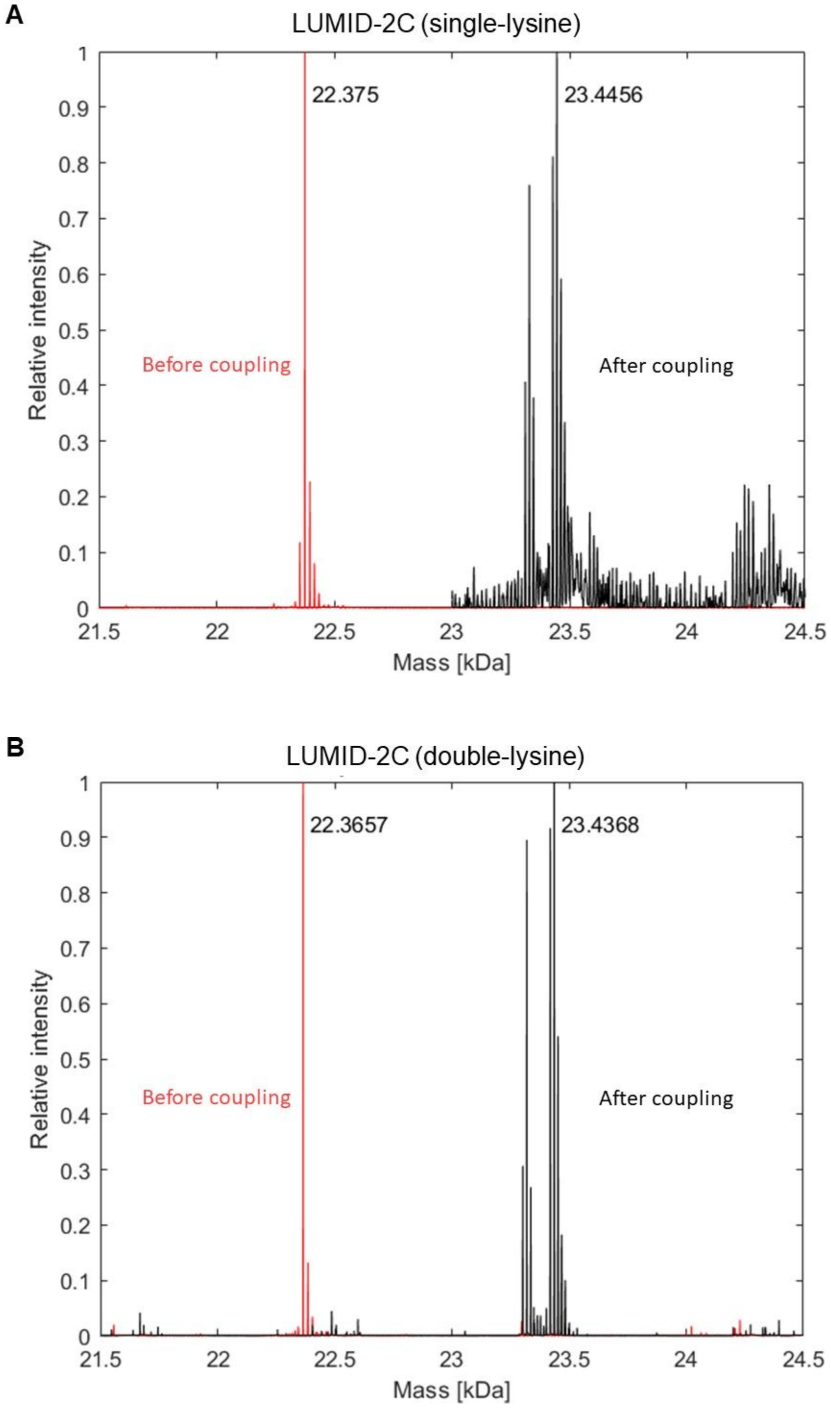

Figure S9 | ESI-QTOF mass spectra of LUMID-2C with single- and double-lysine linker, before and after dye coupling. A LUMID-2C single-lysine (calculated mass without dyes: 22377 Da, with 2 dyes: 23.431 kDa, with 2 dyes and 1 oxidized thiol: 23.447 kDa). **B** LUMID-2C double-lysine (calculated mass without dyes: 22.368 kDa, with 2 dyes: 23.422 kDa, with 2 dyes and 1 oxidized thiol: 23.438 kDa). See supplementary table S2 for molecular weight calculations, including possible modifications.

A

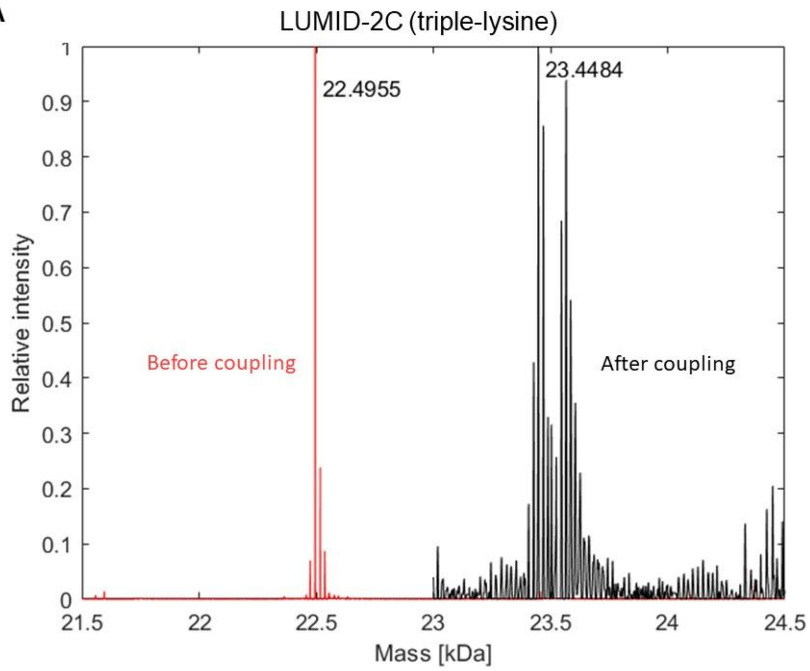

B

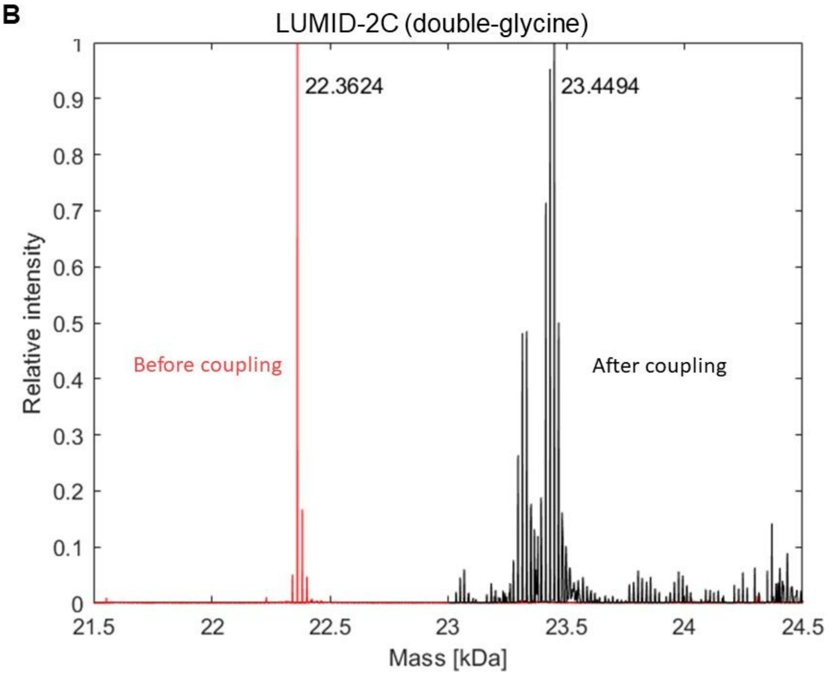

Figure S10 | ESI-QTOF mass spectra of LUMID-2C with triple-lysine and double-glycine linker, before and after dye coupling. A LUMID-2C triple-lysine (calculated mass without dyes: 22.496 Da, with 2 dyes: 23.550 Da). **B** LUMID-2C double-glycine (calculated mass without dyes: 22.363 Da, with 2 dyes: 23.417 Da, with 2 dyes and 2 oxidized thiols: 23.449 Da). See supplementary table S2 for molecular weight calculations, including possible modifications.

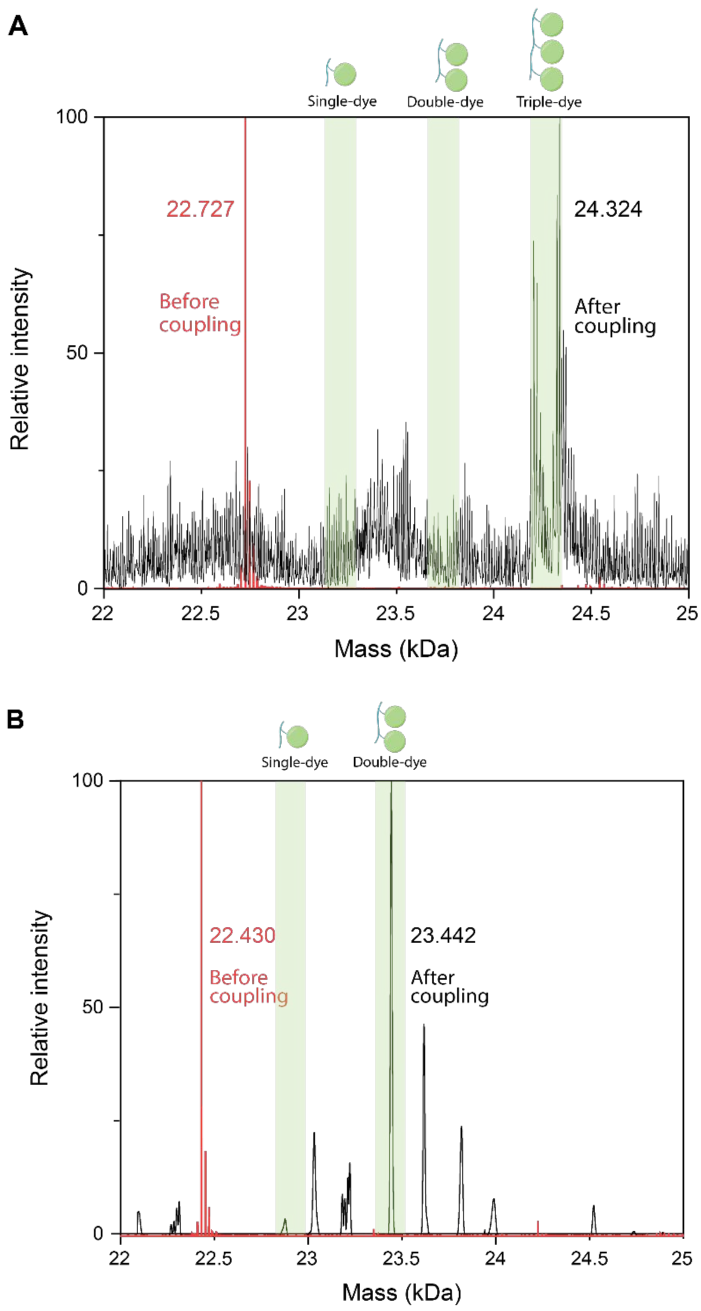

Figure S11 | ESI-QTOF mass spectra of LUMID-3C and LUMID-2F proteins, before and after dye coupling. A LUMID-3C (calculated mass without dyes: 22727 Da, with 3 dyes and 1 oxidized thiol: 23424 Da). **B** LUMID-2F (calculated mass without dyes: 22431 Da, with 2 dyes: 23484 Da). The LUMID-2F mass spectrum after dye coupling is obtained with a high spectrum blur width (>1.0 Da), and therefore only indicative. The exact mass could not be obtained due to large amounts of salts bound to the protein. Areas highlighted in green indicate the expected mass range for sensors containing either one, two or three thiazole orange moieties. The mass range is determined to be distance between the typical two-peak pattern (MW-118 to MW+34), seen for all previous LUMID variants. See supplementary table S2 for molecular weight calculations, including possible modifications.

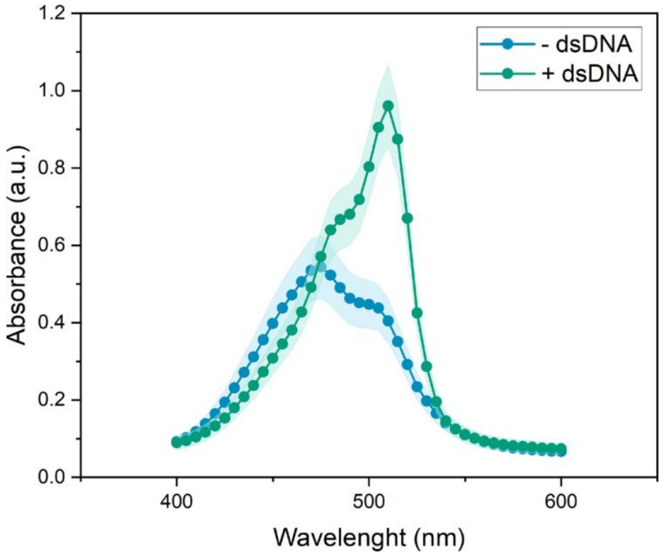

Figure S12 | Absorbance spectrum maleimide-activated Thiazole Orange The absorbance of 1 mM of maleimide-activated Thiazole Orange, in absence (blue) and presence of sheared salmon sperm dsDNA (green, 7.7 mM of base pairs). Data represents mean ± sd, with n=2 technical replicates.

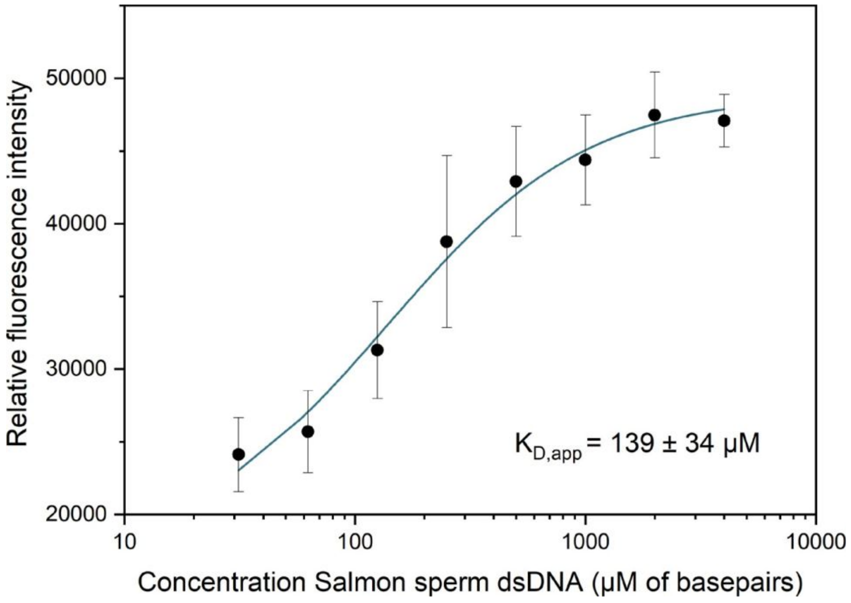

Figure S13 | Titrations of maleimide-activated Thiazole Orange with dsDNA. The dye (1 µM) was added to a 2-fold dilution series of Salmon Sperm DNA ranging from (in base pairs of DNA) 32 µM – 4.000 µM. Incubation was performed at room temperature for 30 minutes in 1xPBS + 1 mg/mL BSA, 5% DMSO, pH 7.4. The fluorescence intensity at 530 nm was measured, using an excitation wavelength of 488 nm. Circles represent mean values ± sd, and solid line represents the fit using a hill function with offset.

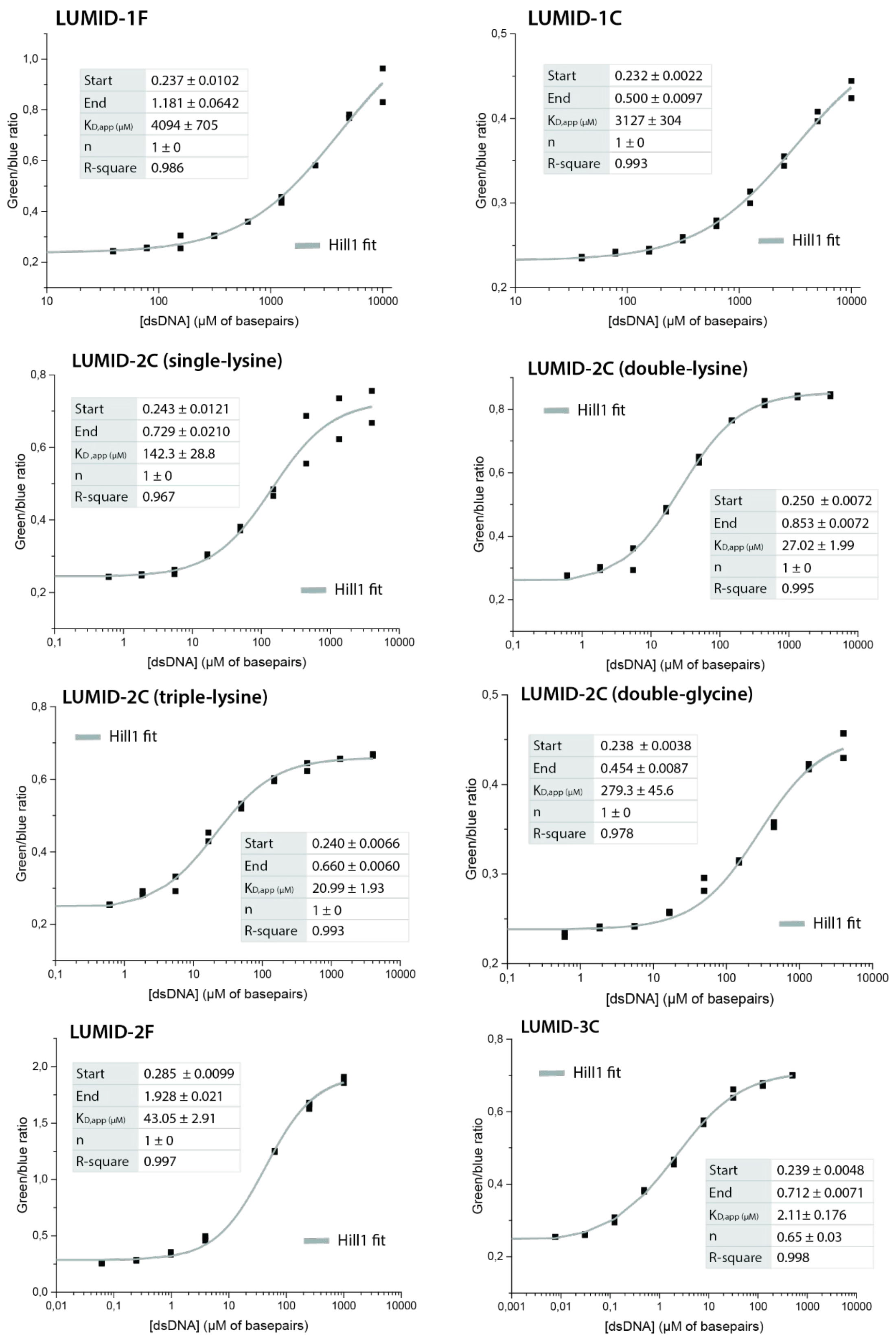

Figure S14 | Model fits of different LUMID variants. Unless stated otherwise, bioluminescence titrations were performed in technical duplicates with n=2 independent preparations of the dsDNA and 1 nM of sensor protein. For LUMID-2F, data represents technical triplicates with n=3 independent preparations of dsDNA. The acquired data was fitted with a Hill function with offset. Insets represent the fitting parameters and R-squared value. For all sensor variants except LUMID-3C the Michaelis Menten constant (n) was fixed to 1.

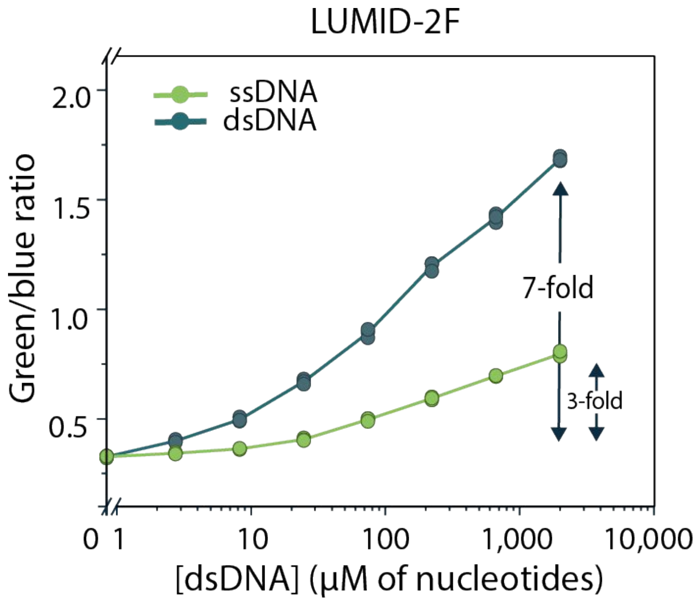

Figure S15 | Titrations LUMID-2F with ssDNA and dsDNA. LUMID-2F (2 nM) was added to a 3-fold dilutions series of oligonucleotide (41 bp, with and without complementary strand) ranging from 2.7 μM – 2,000 μM (nucleotides) and incubated for 30 minutes at room temperature before the addition of substrate. Luminescence was recorded at 458 nm (blue) and 533 nm (green). The fold increase in green/blue ratio is indicated in each graph. Circles represent n = 3 technical replicates, and lines connect mean values.

```
atg tgg tct cat cct caa ttt gaa aaa atg gta ttt act ctt gaa gat ttt gtc ggt gat
 M   W   S   H   P   Q   F   E   K   M   V   F   T   L   E   D   F   V   G   D
                                20                                      30
tgg cgc cag acc gcc ggc tat aac ctg gac caa gtg ctt gaa cag ggc ggg gtt agc agc
 W   R   Q   T   A   G   Y   N   L   D   Q   V   L   E   Q   G   G   V   S   S
                                40                                  50
ctg ttt caa aac ctg ggg gtg agt gtc acg cca att cag cgc atc gtt ctg tcg gga gag
 L   F   Q   N   L   G   V   S   V   T   P   I   Q   R   I   V   L   S   G   E
                                60                                      70
aat ggt ctg aaa atc gat atc cac gtc att atc ccg tac gaa ggt ctt tct ggt gat cag
 N   G   L   K   I   D   I   H   V   I   I   P   Y   E   G   L   S   G   D   Q
                                80                                      90
atg ggg cag ata gaa aaa ata ttc aaa gtg gtg tac cca gta gac gat cat cac ttc aag
 M   G   Q   I   E   K   I   F   K   V   V   Y   P   V   D   D   H   H   F   K
                                100                                     110
gtt ata ctg cac tat ggc acc ctc gtt atc gat ggc gtt act ccg aat atg atc gat tac
 V   I   L   H   Y   G   T   L   V   I   D   G   V   T   P   N   M   I   D   Y
                                120                                     130
ttt ggg cgt cct tat gaa ggt att gcg gtg ttc gac ggt aaa aaa att acg gtt acc ggg
 F   G   R   P   Y   E   G   I   A   V   F   D   G   K   K   I   T   V   T   G
                                140                                     150
acg ctc tgg aat ggt aat aaa atc att gat gag cgc ttg ata aac cca gat ggc agc ctt
 T   L   W   N   G   N   K   I   I   D   E   R   L   I   N   P   D   G   S   L
                                160                                     170
ctg ttc aga gtt acg ata aac ggg gtt acg ggt tgg cga ctg agc gaa aga ata tta gct
 L   F   R   V   T   I   N   G   V   T   G   W   R   L   S   E   R   I   L   A
                                  180
      gcg tcc gca ctc gag ctg cca gaa acc ggt ggt cac cac cac cac cac cac
       A   S   A   L   E   L   P   E   T   G   G   H   H   H   H   H   H
```

Figure S16 | DNA and amino acid sequence of NanoLuc. The native cysteine was mutated to a serine (C166S, yellow). A STREP-tag (pink) and hexahistidine-tag (blue) are incorporated at the N- and C- terminus, respectively. The position D148 (green) is chosen as the conjugation site in the flexible loop region, and the position G182 (red) is chosen as the conjugation site at the C-terminus

TAATACGACTCACTATAGGGGAAAGGTAAGATGGAGAGCCTTGTCCCTGGTTTCAACGAGAAAACACACG
TCCAACTCAGTTTGCCTGTTTTACAGGTTCGCGACGTGCTCGTACGTGGCTTTGGAGACTCCGTGGAGG
AGGTCTTATCAGAGGCACGTCAACATCTTAAAGATGGCACTTGTGGCTTAGTAGAAGTTGAAAAAGGCGT
TTTGCCTCAACTTGAACAGCCCTATGTGTTCATCAAACGTTCGGATGCTCGAACTGCACCTCATGGTCAT
GTTATGGTTGAGCTGGTAGCAGAACTCGAAGGCATTCAGTACGGTCGTAGTGGTGAGACACTTGGTGTC
CTTGTCCCTCATGTGGGCGAAATACCAGTGGCTTACCGCAAGGTTCTTCTTCGTAAGAACGGTAATAAAG
GAGCTGGTGGCCATAGTTACGGCGCCGATCTAAAGTCATTTGACTTAGGCGACGAGCTTGGCACTGATC
CTTATGAAGATTTTCAAGAAAACTGGAACACTAAACATAGCGCTTCGCCTAGGCCGCTGAGCAATAACTA
GCATAACCCCTTGGGGCCTCTAAACGGGTCTTGAGGGGTTTTTTGCTGAAAACCTCGCTCGCTGAGGTG
TCAATCGTCGGAGCCGCTGAGCAATAACTAGCATAACCCCTTGGGGCCTCTAAACGGGTCTTGAGGGGT
TTTTTGCATGGTCATAGCTGTTTCCTG

Figure S17 | Sequence target DNA of the SARS-CoV-2 ORF1a complementary DNA. Sequence is presented as 5’ → 3’

TAATACGACTCACTATAGGGATGTCTGATAATGGACCCCAAAATCAGCGAAATGCACCCCGCATTACGTT
TGGTGGACCCTCAGATTCAACTGGCAGTAACCAGAATGGAGAACGCAGTGGGGCGCGATCAAAACAACG
TCGGCCCCAAGGTTTACCCAATAATACTGCGTCTTGGTTCACCGCTCTCACTCAACATGGCAAGGAAGAC
CTTAAATTCCCTCGAGGACAAGGCGTTCCAATTAACACCAATAGCAGTCCAGATGACCAAATTGGCTACT
ACCGAAGAGCTACCAGACGAATTCGTGGTGGTGACGGTAAAATGAAAGATCTCAGTCCAAGATGGTATTT
CTACTACCTAGGAACTGGGCCAGAAGCTGGACTTCCCTATGGTGCTAACAAAGACGGCATCATATGGGTT
GCAACTGAGGGAGCCTTGAATACACCAAAAGATCACATTGGCACCCGCAATCCTGCTAACAATGCTGCAA
TCGTGCTACAACTTCCTCAAGGAACAACATTGCCAAAAGGCTTCTACGCAGAAGGGAGCAGAGGCGGCA
GTCAAGCCTCTTCTCGTTCCTCATCACGTAGTCGCAACAGTTCAAGAAATTCAACTCCAGGCAGCAGTAG
GGGAACTTCTCCTGCTAGAATGGCTGGCAATGGCGGTGATGCTGCTCTTGCTTTGCTGCTGCTTGACAG
ATTGAACCAGCTTGAGAGCAAAATGTCTGGTAAAGGCCAACAACAACAAGGCCAAACTGTCACTAAGAAA
TCTGCTGCTGAGGCTTCTAAGAAGCCTCGGCAAAAACGTACTGCCACTAAAGCATACAATGTAACACAAG
CTTTCGGCAGACGTGGTCCAGAACAAACCCAAGGAAATTTTGGGGACCAGGAACTAATCAGACAAGGAA
CTGATTACAAACATTGGCCGCAAATTGCACAATTTGCCCCCAGCGCTTCAGCGTTCTTCGGAATGTCGCG
CATTGGCATGGAAGTCACACCTTCGGGAACGTGGTTGACCTACACAGGTGCCATCAAATTGGATGACAAA
GATCCAAATTTCAAAGATCAAGTCATTTTGCTGAATAAGCATATTGACGCATACAAAACATTCCCACCAAC
AGAGCTTCGCCTAGGCCGCTGAGCAATAACTAGCATAACCCCTTGGGGCCTCTAAACGGGTCTTGAGGG
GTTTTTTGCTGAAAACCTCGCTCGCTGAGGTGTCAATCGTCGGAGCCGCTGAGCAATAACTAGCATAACC
CCTTGGGGCCTCTAAACGGGTCTTGAGGGGTTTTTTGCATGGTCATAGCTGTTTCCTG

Figure S18 | Sequence of the SARS-CoV-2 nucleocapsid gene complementary DNA (ORF_N). Sequence is presented as 5’ → 3’

Supplementary tables

table S1 | Mutation sites of the different NanoLuc variants. 1), 2) and 3) indicate that mutagenesis was performed in multiple steps, combining point mutations and insertions. The colors indicate the positions of the mutations as seen in supplementary Figure S16.

NanoLuc variant	Additional mutation sites
1 dye at the C-terminus	G182C
2 dyes at the C-terminus, 1 lysine linker	1) G182C<br>2) Insertion: ETGCHHH -> ETGCKCHHH
2 dyes at the C-terminus, 2 lysine linker	1) G182C<br>2) Insertion: ETGCHHH -> ETGCKKCHHH
2 dyes at the C-terminus, 3 lysine linker	1) G182C<br>2) Insertion: ETGCHHH -> ETGCKKKCHHH
3 dyes at the C-terminus, 2 lysine linker	1) G182C<br>2) Insertion: ETGCHHH -> ETGCKKCHHH<br>3) Insertion: ETGCKKCHHH -> ETGCKKCKKCHHH
1 dye at the flexible loop	D148C
2 dyes at the flexible loop	1) D148C<br>2) Insertion: INPCGSL -> INPCKKCGSL

table S2 | Calculated molecular weights for the different NanoLuc constructs and modifications, including: without dyes, with dyes and with hydrolyzed maleimide functionalities or oxidized thiols[2]**.**

Construct	MW protein	MW dye(s)	MW protein + dye(s)	Maleimide hydrolysis (+18)	Oxidation Thiol* (+16)	Hydrolysis + oxidation* (+34)
NL1F	22071.3	572.2	22598.5	22616.5	22614.5	22632.5
NL1C	22145.3	572.2	22672.5	22690.5	22688.5	22706.5
NL2C-1K	22376.6	1054.4	23431.0	23449 (1x)<br>23467 (2x)	23447(1x)<br>23463(2x)	23465
NL2C-2K	22367.6	1054.4	23422.0	23440 (1x)<br>23458 (2x)	23438 (1x)<br>23454 (2x)	23456
NL2C-3K	22495.9	1054.4	23550.3	23568.3 (1x)<br>23586.3 (2x)	23566.3 (1x)<br>23582.3 (2x)	23584.3
NL2C-2G	22362.6	1054.4	23417.0	23435 (1x)<br>23453 (2x)	23433 (1x)<br>23449 (2x)	23451
NL2F-2K	22430.8	1054.4	23484.4	23502.4 (1x)<br>23520.4 (2x)	23500.4 (1x)<br>23516.4 (2x)	23518.4
NL3C-2K	22727.2	1581.6	24308.8	24326.8 (1x)<br>24344.8 (2x)<br>24362.8 (3x)	24324.8 (1x)<br>24340.8 (2x)<br>24356.8 (3x)	24342.8

**Includes also additional +16 adducts, up to two oxidations per thiol moiety.*

*** Includes also additional +16 and +18 adducts.*

table S3 | Sequences of the primers used for mutagenesis.

Construct	Short description	Sequence (5' → 3')
NL	Used for mutating the native cysteine to a serine (C164S)	CGAGCTGCCAGAAACCGGTTGTCA CCACCACCACCACCACTG
NL1C	Used for the G182C mutation	GGGTTACGGGTTGGCGACTGAGCG AAAGAATATTAGCTGCGGCCGC
NL1F	Used for the D148C mutation	AGCGCTTGATAAACCCA TGT GGCAGCCTTCTGTTCAG
NL2C-1K	Used for insertion of KC downstream the cysteine present in NL1C	CAGAAACCGGTTGCAAATGTCACC ACCACCACC
NL2C-2K	Used for insertion of KKC downstream the cysteine present in NL1C	CAGAAACCGGTTGTAAAAAATGCCA CCACCACCACC
NL2C-3K	Used for insertion of KKKC downstream the cysteine present in NL1C	CAGAAACCGGTTGTAAAAAAAAGTG CCACCACCACC
NL2C-2G	Used for insertion of GGC downstream the cysteine present in NL1C.	CAGAAACCGGTTGCGGCGGCTGTC ACCACCACCACC
NL3C	Used for insertion of KKC, downstream the cysteine present in NL1F	CCAGAAACCGGTTGCAAGAAGTGT AAAAAATGCCACCACC
NL2F	Used for insertion of KKC, downstream the cysteine present in NL2C-2K	GCTTGATAAACCCATGTAAAAAATG CGGCAGCCTTCTGTTC

table S4 | Sequences of the RPA primers targeting ORF_1a (supplementary Figure S17). Primers were designed using the PrimedRPA software, as described in Higgins et al.[3]

Primer name	Sequence (5' → 3')
Forward	GCTGGTAGCAGAACTCGAAGGCATTCAGTAC
Reverse	ACCAGCTCCTTTATTACCGTTCTTACGAAGA

table S5 | Sequences of the RPA primers targeting ORF_N (supplementary Figure S18). Primers were designed using the PrimedRPA software, as described in Higgins et al.[3]

Primer name	Sequence (5' → 3')
Set 1	
Forward 1	CAGTAACCAGAATGGAGAACGCAGTGGGGCG
Reverse 1	ACGTGATGAGGAACGAGAAGAGGCTTGACTG
Set 2	
Forward 2	ATAATACTGCGTCTTGGTTCACCGCTCTCAC
Reverse 2	GTGCCAATGTGATCTTTTGGTGTATTCAAGG
Set 3	
Forward 3	CGTTCCAATTAACACCAATAGCAGTCCAGAT
Reverse 3	AAGCAGCAGCAAAGCAAGAGCAGCATCACCG

table S6 | Sequences of the LAMP primers targeting ORF_N (supplementary Figure S18) Primers were designed using the NEB LAMP primer design tool.

Primer name	Sequence (5' → 3')
Set 1	
Forward inner primer	TCCCCTACTGCTGCCTGGAGGCAGTCAAGCCTCTTCTCG
Backward inner primer	TCTCCTGCTAGAATGGCTGGCATCTGTCAAGCAGCAGCAAAG
Forward outer primer	GCCAAAAGGCTTCTACGCA
Backward outer primer	TTGCTCTCAAGCTGGTTCAA
Forward loop	GCGACTACGTGATGAGGAA
Backward loop	GGCGGTGATGCTGCTCTT
Set 2	
Forward inner primer	CCACTGCGTTCTCCATTCTGGTAAATGCACCCCGCATTACG
Backward inner primer	CGCGATCAAAACAACGTCGGCCCTTGCCATGTTGAGTGAGA
Forward outer primer	TGGACCCCAAAATCAGCG
Backward outer primer	GCCTTGTCCTCGAGGGAAT
Forward loop	TTGAATCTGAGGGTCCACC
Backward loop	GGTTTACCCAATAATACTGCGTCTT
Set 3	
Forward inner primer	TCTGCGTAGAAGCCTTTTGGCACAATGCTGCAATCGTGCTAC
Backward inner primer	GGCGGCAGTCAAGCCTCTTCCTACTGCTGCCTGGAGTTG
Forward outer primer	AGATCACATTGGCACCCG
Backward outer primer	CCATTGCCAGCCATTCTAGC
Forward loop	TGTTGTTCCTTGAGGAAGTT
Backward loop	TCGTTCCTCATCACGTAGTCG

Supplementary references

1. Chester, N. & Marshak, D. R. Dimethyl sulfoxide-mediated primer Tm reduction: A method for analyzing the role of renaturation temperature in the polymerase chain reaction. *Analytical Biochemistry* **209**, 284–290 (1993).
2. Boyatzis, A. E. *et al.* Limiting the Hydrolysis and Oxidation of Maleimide-Peptide Adducts Improves Detection of Protein Thiol Oxidation. *J. Proteome Res.* **16**, 2004–2015 (2017).
3. Higgins, M. *et al.* PrimedRPA: Primer design for recombinase polymerase amplification assays. *Bioinformatics* **35**, 682–684 (2019).
